# Global Axial Length Centile Charts

**DOI:** 10.1001/jamaophthalmol.2026.2539

**Published:** 2026-07-16

**Authors:** Sander C. M. Kneepkens, Gareth Lingham, Dirk J. van Hemert, James Loughman, Niall C. Strang, Yih Chung Tham, Siofra C. Harrington, Laura Guisasola, Amanda French, Xiangui He, David A. Mackey, Lene A. Hagen, Rigmor C. Baraas, Olavi Pärssinen, Jason C. Yam, Kathryn J. Saunders, Jeremy A. Guggenheim, Seang Mei Saw, Weizhong Z. Lan, Caroline C. W. Klaver, D. Ian Flitcroft

**Affiliations:** 1 Department of Ophthalmology, Erasmus University Medical Center, Rotterdam, the Netherlands; 2 The Generation R Study Group, Erasmus University Medical Center, Rotterdam, the Netherlands; 3 Centre for Ophthalmology and Visual Science (incorporating the Lions Eye Institute), University of Western Australia, Perth, Western Australia, Australia; 4 Centre for Eye Research Ireland, Environmental, Sustainability and Health Institute, Technological University Dublin, Dublin, Ireland; 5 Centre for Eye Research Australia, Melbourne, Victoria, Australia; 6 Department of Vision Sciences, Glasgow Caledonian University, Glasgow, United Kingdom; 7 Singapore Eye Research Institute, Singapore National Eye Centre, Singapore, Singapore; 8 School of Physics, Clinical & Optometric Sciences, Technological University Dublin, Dublin, Ireland; 9 Visió Optometria i Salut, Department of Optics and Optometry, Universitat Politècnica de Catalunya, Barcelona, Spain; 10 Discipline of Orthoptics, University of Technology Sydney, Sydney, New South Wales, Australia; 11 Shanghai Eye Diseases Prevention & Treatment Center, Shanghai Eye Hospital, School of Medicine, Tongji University, Shanghai, China; 12 National Centre for Optics, Vision and Eye Care, University of South-Eastern Norway, Kongsberg, Norway; 13 Department of Ophthalmology, Central Hospital of Central Finland, Jyväskylä, Finland; 14 Gerontology Research Centre and Faculty of Sport and Health Sciences, University of Jyväskylä, Jyväskylä, Finland; 15 Department of Ophthalmology and Visual Sciences, The Chinese University of Hong Kong, Hong Kong SAR, China; 16 Hong Kong Eye Hospital, Hong Kong SAR, China; 17 Centre for Optometry and Vision Science, Biomedical Sciences Research Institute, Ulster University, Coleraine, United Kingdom; 18 School of Optometry & Vision Sciences, Cardiff University, Cardiff, United Kingdom; 19 Ophthalmology and Visual Science Academic Clinical Program, Duke-NUS Medical School, Singapore, Singapore; 20 Saw Swee Hock School of Public Health, National University of Singapore, Singapore, Singapore; 21 Aier Academy of Ophthalmology, Central South University, Changsha, China; 22 Aier School of Optometry and Vision Science, Hubei University of Science and Technology, Xianning, China; 23 Department of Epidemiology, Erasmus University Medical Center, Rotterdam, the Netherlands; 24 Department of Ophthalmology, Radboud University Medical Center, Nijmegen, the Netherlands; 25 Institute of Molecular and Clinical Ophthalmology, Basel, Switzerland; 26 Mater Misericordiae Hospital, Dublin, Ireland

## Abstract

**Importance:**

Myopia is increasingly common worldwide and poses major public health challenges because axial elongation drives sight-threatening complications. Because axial length (AL) is the primary target for myopia management, population-specific AL reference charts are needed.

**Objective:**

To generate region- and sex-specific reference centile charts for AL in children and adolescents.

**Design, Setting, and Participants:**

This was a pooled cohort analysis using individual-participant data from population- and school-based studies conducted in East Asia, Europe, and Australia contributing to the CREAM-Kids Consortium. A total of 147 404 children and adolescents contributing 559 799 AL measurements were included. Most participants were from East Asia (84%; ages 6-18 years), followed by Europe (12%; ages 6-21 years) and Australia (4%; ages 6-21 years). After assessment of comparability, European and Australian datasets were combined. Data were included from 1999 to 2024, with the analysis taking place from December 2024 to May 2026.

**Exposures:**

AL, age, sex, and geographic region.

**Main Outcomes and Measures:**

Region- and sex-specific AL centiles across age and agreement between modeled centiles and empirical values.

**Results:**

AL distributions among the 147 404 included individuals varied by sex and region. In female individuals, the 50th centile AL at age 7 years was 22.35 mm in Europe and Australia and 22.67 mm in East Asia; by age 18 years, the 50th centile ALs were 23.31 mm and 24.36 mm, respectively. Regional differences were larger at higher centiles: for female individuals at age 7 years, East Asia vs Europe and Australia differences were 0.33 mm at the 3rd centile and 0.50 mm at the 97th centile; at age 18 years, differences were 0.73 mm and 1.27 mm, respectively. Modeled centile curves closely matched empirical values (intraclass correlations >0.99) with minimal bias across sex and region subgroups (–0.08 mm to 0.04 mm).

**Conclusions and Relevance:**

While not based solely on population-based cohorts, the region- and sex-specific AL centile charts developed in this pooled cohort study provide reference values for childhood and adolescence that may be used to support clinical and research applications, including identifying atypical axial elongation and informing targeted strategies for myopia prevention and management.

## Key Points

**Question:**

How do region-specific axial length (AL) centile curves describe and compare AL distributions across populations, and at what ages do regional differences emerge?

**Findings:**

This pooled cohort study used individual-level data from 147 404 children and 559 799 AL measurements to develop AL centile charts for East Asia and Europe and Australia. East Asian children had longer AL across all centiles; the East Asian 50th percentile approximated the Europe/Australia 85th, with faster early elongation and earlier plateau.

**Meaning:**

These curves could serve as the current reference standard for these regions, supporting interpretation of childhood AL and regional growth trajectories without defining diagnostic thresholds for myopia.

## Teaser

This pooled cohort study draws on data from the CREAM-Kids Consortium to generate global axial length centile charts for children and adolescents by sex and region.

## Introduction

Myopia is increasing in both prevalence and severity worldwide, having already reached epidemic levels in some regions. Global predictions estimate that by 2050, half of the world’s population will have myopia, and at least 10% will have high myopia.^1^ While similar upward trends are evident across all continents, myopia prevalence is currently highest in parts of East and Southeast Asia.^1^ The reported prevalence of myopia among young European adults is 56.0%, compared with 61.5% of 12-year-olds in Hong Kong and 86.1% of young adults in Taiwan.^2,3^

Although myopia can be optically corrected with glasses or contact lenses,^4^ refractive correction does not address the underlying anatomical changes associated with the condition. The onset of myopia is primarily driven by axial elongation of the eye, and further elongation as the condition progresses induces lasting structural changes that can lead to permanent, sight-threatening complications later in life.^5^ The longer the eye, the higher the risk of these complications.^5^ Individuals with high myopia have a one-in-three risk of developing severe visual impairment in both eyes, and those with an axial length (AL) greater than 30 mm have a lifetime risk exceeding 90%.^6^ This poses a substantial public health burden for multiple reasons.^7^ Preventing or delaying myopia onset in childhood is increasingly recognized as a public health priority.^8,9^ Treatments aim to prevent myopia onset and progression by limiting axial eye growth. AL has become the preferred target outcome in myopia management due to its higher precision and stronger link to future risk of pathological complications.^10^

In this study, we constructed region-specific AL centile curves to compare distributions across different populations, drawing on the principles used in height and weight centile charts. These curves describe how AL is distributed within each region, illustrating the spread, skew, and relative position of values across ages. Comparison of centile patterns between regions allows identification of shifts in AL distribution and provides insight into the age at which differences begin to emerge. Our approach provides a structured framework for understanding how AL varies between populations, without implying any specific threshold of normal or healthy development. These are population centile charts, not growth standards, and therefore describe population variation rather than normative or healthy development.

## Methods

### Studies and Participants

This study uses data from the Consortium of Refractive Error and Myopia Research in Children (CREAM-Kids), an international collaboration combining ophthalmologic and lifestyle data from 20 pediatric cohort studies across Asia, Europe, and Australia. The consortium includes both school-based and population-based studies and has a total sample of 172 472 children. Most (14/20) of the collaborating cohorts have a longitudinal design. In this analysis, we included studies with participants aged 6 to 21 years and available data on age, sex, and AL in at least 1 eye (16/20). Participating studies and cohort descriptions are presented in Table 1. Individual study design and sampling methods are provided in the eMethods in Supplement 1. All included cohorts had obtained prior ethical approval and informed consent. The present study used anonymized, previously approved data; therefore, no additional ethical approval was required. Further details for each study are provided in the eMethods 1 in Supplement 1.

**Table 1.** Description of the 16 Consortium Studies Included in This Study, Stratified by Region

Study, country	Data collection period	Urban vs rural	Participants, No.	Observations, No.	Median (IQR)		
					Age, y	Axial length, mm	
						Right eye	Left eye
**European cohorts**							
Avon Longitudinal Study of Parents and Children (ALSPAC), United Kingdom	2006-2008	Mixed	2769	5497	15 (15-16)	23.4 (22.86-23.95)	23.35 (22.83-23.92)
Cohort Infantil de Salut Visual de Terrassa (CISViT) Project, Spain^a^	2020-2024	Urban	1700	5203	8 (8-9)	22.96 (22.41-23.49)	22.9 (22.37-23.47)
Finnish conscripts, Finland	2023	Mixed	1302	2602	19 (18-19)	23.8 (23.26-24.47)	23.67 (23.17-24.30)
Generation R, the Netherlands^a^	2008-2024	Urban	7177	39 760	10 (6-14)	23.03 (22.40-23.69)	23.01 (22.38-23.67)
Glasgow Caledonian Study, Scotland^a^	2006-2010	Urban	140	588	14 (11-17)	23.3 (22.63-24.19)	23.21 (22.59-24.14)
Ireland Eye Study, Ireland	2015-2018	Mixed	1617	3229	12 (7-13)	23.06 (22.41-23.65)	23.06 (22.38-23.69)
Northern Ireland Childhood Errors of Refraction Study, Northern Ireland^a^	2006-2016	Mixed	1047	4734	13 (10-16)	23.2 (22.59-23.84)	23.19 (22.60-23.80)
Refractive Error and Color Vision (RECV) Study, Norway	2015-2016	Mixed	438	876	16 (16-17)	23.44 (22.88-24.05)	23.42 (22.91-24.05)
Southeast Norway Vision and Visuomotor (SNOW) Study, Norway	2015-2024	Mixed	1360	2710	15 (9-15)	23.11 (22.55-23.70)	23.07 (22.52-23.68)
**East Asian cohorts**							
Growing up in Singapore Toward Healthy Outcomes (GUSTO) Study, Singapore	2015-2017	Urban	483	1384	9 (6-9)	23.03 (22.46-23.77)	23.03 (22.47-23.78)
Hong Kong Children Eye Study, China	2014-2021	Urban	21 197	42 199	8 (7-8)	23.1 (22.51-23.73)	23.1 (22.50-23.72)
Shanghai Time Outside to Reduce Myopia (STORM), China	2016-2018	Urban	6463	12 926	9 (7-12)	23.56 (22.85-24.46)	23.53 (22.83-24.40)
Singapore Cohort of Risk Factors for Myopia (SCORM), Singapore^a^	1999-2004	Urban	1967	7175	9 (8-11)	23.52 (22.86-24.29)	23.5 (22.86-24.30)
West China Refractive Error Development Study, China^a^	2018-2022	Urban	94 376	416 335	9 (7-11)	23.5 (22.82-24.31)	23.46 (22.79-24.24)
**Australian cohorts**							
Raine Study Gen2, Australia	2010-2020	Urban	1298	2596	20 (20-20)	23.53 (23.01-24.13)	23.5 (22.99-24.09)
Sydney Myopia Study, Australia^a^	2003-2012	Urban	4070	11 985	13 (7-13)	23.17 (22.62-23.75)	23.16 (22.60-23.73)

^a^ Indicates a cohort contributing longitudinal data.

### Measurements and Data Collection

Individual-level data from each participating cohort were collected and harmonized into a unified CREAM-Kids dataset. AL measurements were obtained with optical biometry devices using optical interferometry, such as the IOLMaster 500/700 (Carl Zeiss Meditec AG) and Lenstar LS 900 (Haag-Streit AG) or the A-scan biometry machine (the eMethods in Supplement 1). Ethnicity-specific centile charts were not developed for 2 reasons: (1) the effect of ethnicity on AL likely varies across regions,^11^ and (2) the centile charts are intended for universal use within a region, without being complicated by assessment of an individual’s ethnicity. This approach aligns with the Multicenter Growth Reference Study from the World Health Organization (WHO).^12^

### Statistical Analysis

AL measurements below 15 mm or above 35 mm were considered invalid and likely due to measurement error. Measurements from both eyes from all visits were included in the analysis. As no participant was examined twice at the same age, each participant could contribute up to 2 observations per age-bin (1 each from the right and left eye). Inclusion of correlated data points (such as 2 eyes from an individual) can lead to underestimation of the true variance but does not bias the estimates themselves.^13^ Where variance was estimated, we accounted for correlated data using linear mixed models; primary data were available for all studies; however, due to data-sharing constraints, the Shanghai Time Outside to Reduce Myopia (STORM) study data and a subset (43.1%) of the Generation R data could not be pooled with other datasets. These cohorts provided the required summary statistics generated locally using harmonized protocols. To moderate the influence of very large studies, the square-root-weighting approach was applied. Given that AL distributions are typically right-skewed, especially in older children, we used nonparametric statistical methods.

### Pooling Studies

To evaluate the validity of pooling data, we compared between-study variance estimates within and across European, Australian, and East Asian cohorts. Variances were estimated from linear mixed models using AL of the right eye as the outcome variable, to avoid variance attributable to systematic right vs left eye differences.^14^ Fixed effects included age, the natural logarithm of age, and sex. Random intercept effects were participant identification number nested within each study, allowing estimation of variance attributable to between-study differences, individual-level differences, and residual error. We did not adjust for biometry device or calendar year of studies because these variables clustered by cohort and were thus collinear. Between-study variance was examined when studies within a region were pooled and when studies across regions were pooled. In alignment with the WHO infant growth charts methodology, a between-study variance greater than 3% was considered too large to justify pooling.^15^

### Generating AL Centiles

To generate the centile models, we followed methods we previously established for analyzing refractive error data.^16^ These methods do not follow the typical, single-step analysis using generalized additive models for location, scale and shape (GAMLSS), but they do follow similar principles in that they (1) model the distribution and then (2) estimate smoothed associations between age and the fitted model quantiles (GAMLSS estimates relationships with model parameters but the outcome is similar).^17^ A key advantage of our method is that the centile model can be fitted from summary data, preventing the need to share raw data, which may be prohibited by local data protection laws.

We first extracted empirical centile (or quantile) values stratified by age and sex from each study. Age was grouped into 1-year intervals; however, to ensure stability of centile estimates, subgroups with fewer than 85 participants^18^ were merged for up to 3 consecutive years of age, and the mean age of the combined group was used for modeling. This threshold was based on prior meta-analytic standards.^12^ All observations from each participant were used, although each observation was used only once. A multi-Gaussian model was then fitted to these empirical centile values—first attempting a 2-Gaussian fit, which reverted to a single Gaussian fit if the model failed to converge—and fitted centile values were reextracted to improve consistency across subgroups of varying sample sizes. Multi-Gaussian models were chosen because we have previously shown they better fit refractive error distributions compared to the WHO-recommended Box-Cox Power Exponential. To validate this approach with AL distributions, which tend to be less skewed and less kurtotic than refractive error distributions, we fitted raw AL distribution data using multi-Gaussian models, the Box-Cox Power Exponential and using the normal distribution after a square root transformation. Fitted model distributions were compared to the raw distributions by calculating the area between the empirical and fitted cumulative density functions and the Wasserstein distance, a measure of the difference between 2 cumulative density functions.

Final centile curves were generated by fitting weighted cubic splines to the multi-Gaussian model-derived centile values as a function of the logarithm of age, stratified by sex and region. To avoid disproportionate influence from very large studies, weights were assigned based on the square root of the number of observations (eyes) within each age and sex-specific subgroup. In line with the WHO recommendations for infant growth charts, the Generalized Akaike Information Criterion (GAIC) was used to balancing smoothness with goodness of fit by searching the range between 2 and 6 degrees of freedom in 0.025 increments to find the spline curve with the lowest Generalized Akaike Information Criterion for each centile.^12^ Because ALs do not decrease with age, fitted splines were forced to be monotonically increasing to ensure biological plausibility. Uncertainty estimates for centiles were generated using bootstrap resampling clustered within study, sex, and age (1-year intervals) groups with 1000 replicates. Centile models were fit to each replicate sample and the 95% CI for centile curves calculated using the percentile method.

### Evaluating Centile Models

Model performance was evaluated by comparing the fitted centile values to empirical centiles derived from pooled primary data within each region, stratified by age, sex, and region. Agreement between model and empirical centiles was assessed using Bland-Altman statistics, worm plots, and the proportion of pooled sample observations that were at or below each centile value in the fitted model. These evaluate the internal consistency of the model—how well it reflects the underlying data—rather than representing external validation. We also conducted a leave-1-cohort-out analysis by recreating centile models excluding 1 study at a time and calculating the mean absolute deviation across the 3rd, 15th, 50th, 85th, and 97th centiles for both male and female individuals. All statistical analyses were conducted in R version 4.3.2 (R Foundation for Statistical Computing). The results of these analyses are reported in the eResults in Supplement 1.

### Sensitivity Analyses

As sensitivity analyses, we fit a more typical single-step GAMLSS model using the available raw data (ie, excluding STORM and some Generation R data). We used the gamlss package in R to fit GAMLSS models using the logarithm of age as the explanatory variable and forcing the location parameter to be monotonically increasing (as AL increases with age). To investigate any potential influence from including data from both eyes, we also reran the current two-step model restricting data to one eye per visit and one eye per person across all visits. The results of these analyses are reported in the eResults in Supplement 1.

## Results

### Demographic Characteristics

After exclusions, 559 799 AL observations from 147 404 participants were analyzed. Most participants were from East Asia (n = 124 486; 84%) followed by Europe (n = 17 550; 12%) and Australia (n = 5368; 4%). The number of AL observations mirrored this distribution, with 480 019 (86%) from East Asia, 65 199 (12%) from Europe, and 14 581 (3%) from Australia. AL values ranged from 16.11 mm to 30.16 mm. Demographic and ocular characteristics by study are summarized in Table 1. Sex distributions were comparable across the 3 regions, with a slight male predominance in all regions (East Asia: 52.0%, Australia: 50.5%, and Europe: 53.3%).

### Pooling Cohorts

An analysis of median AL demonstrated notable similarities between European and Australian populations, but which visually differed from the East Asian population (eFigure 1 in Supplement 1). Table 2 outlines the variance attributed to the random intercept terms in fitted linear mixed models combining cohorts within and across regions. The between-study variance within each region was consistently low (<1.25%). A leave-1-out sensitivity analysis (eTable 1 in Supplement 1) assessed the influence of individual studies on within-region variance estimates. Notably, exclusion of the Generation R Study reduced between-study variance from 1.21% to 0.63%, while exclusion of the West China Refractive Error Development Study increased between-study variance from 0.27% to 0.97%, indicating a large influence of these cohorts on within-region heterogeneity.

**Table 2.** Comparison of Model Variance Attributed to Between-Study vs Between-Participant Effects When Pooling Within and Across Regions

Region	Variance estimate (% of total variance)		
	Between-study	Between-participant	Residual
Australia	0.004 (0.61)	0.61 (92.13)	0.05 (7.26)
East Asia	0.002 (0.25)	0.74 (92.54)	0.06 (7.21)
Europe	0.009 (1.21)	0.66 (87.50)	0.08 (11.30)
Australia and Europe	0.008 (1.07)	0.64 (87.93)	0.08 (11.00)
Australia and East Asia	0.40 (33.68)	0.73 (61.23)	0.06 (5.10)
East Asia and Europe	0.15 (16.21)	0.73 (76.70)	0.07 (7.08)
Australia and East Asia and Europe	0.14 (14.74)	0.73 (78.00)	0.07 (7.28)

When combining Australian and European cohorts, the between-study variance remained low (1.07%). However, when East Asian studies were pooled with Australian studies, European studies or both, the between-study variance increased substantially to 15% to 34%. Therefore, we decided to pool the cohorts from Australia and Europe.

**Figure 1.**
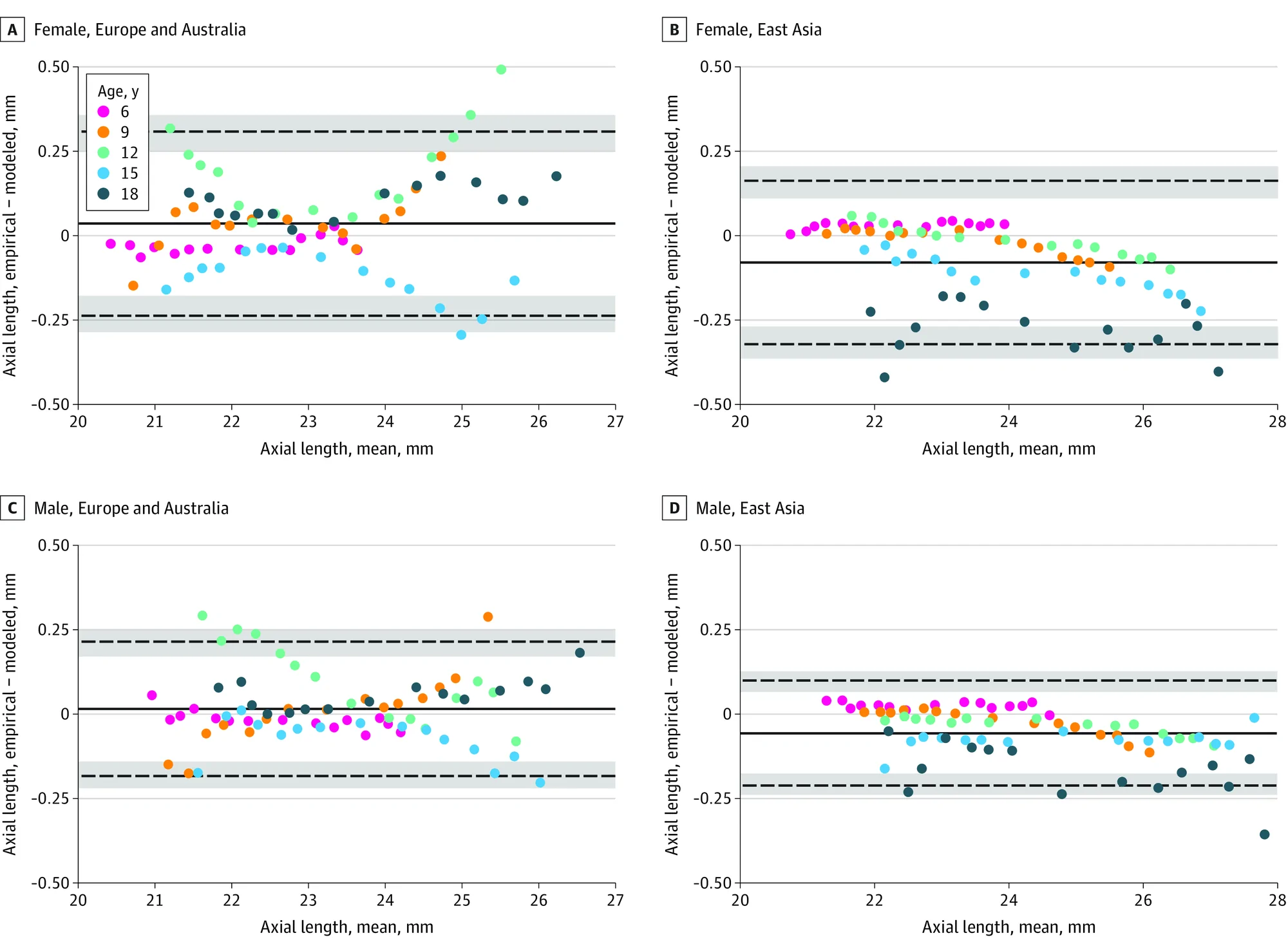
Axial Length Centile Charts Shaded areas represent 95% CIs.

To evaluate the internal consistency of the generated AL growth models, we compared the AL centile values from the model to the empirical AL centile values. The modelled and empirical AL centiles showed good agreement, with intraclass correlations >0.99 for all sex and region categories (Figure 1; eResults in Supplement 1).

### AL Distribution Fitting

eFigure 2 in Supplement 1 demonstrates the multi-gaussian model distribution fit compared to the Box-Cox Power Exponential and square root transform fitting methods, multi-Gaussian fitting of the AL distribution resulted in a closer match to the empirical distribution (median Wasserstein distance: 0.016, 0.022, 0.044 for multi-Gaussian, Box-Cox Power Exponential and square root transform, respectively), although all methods performed reasonably.

### AL Centiles

**Figure 2.**
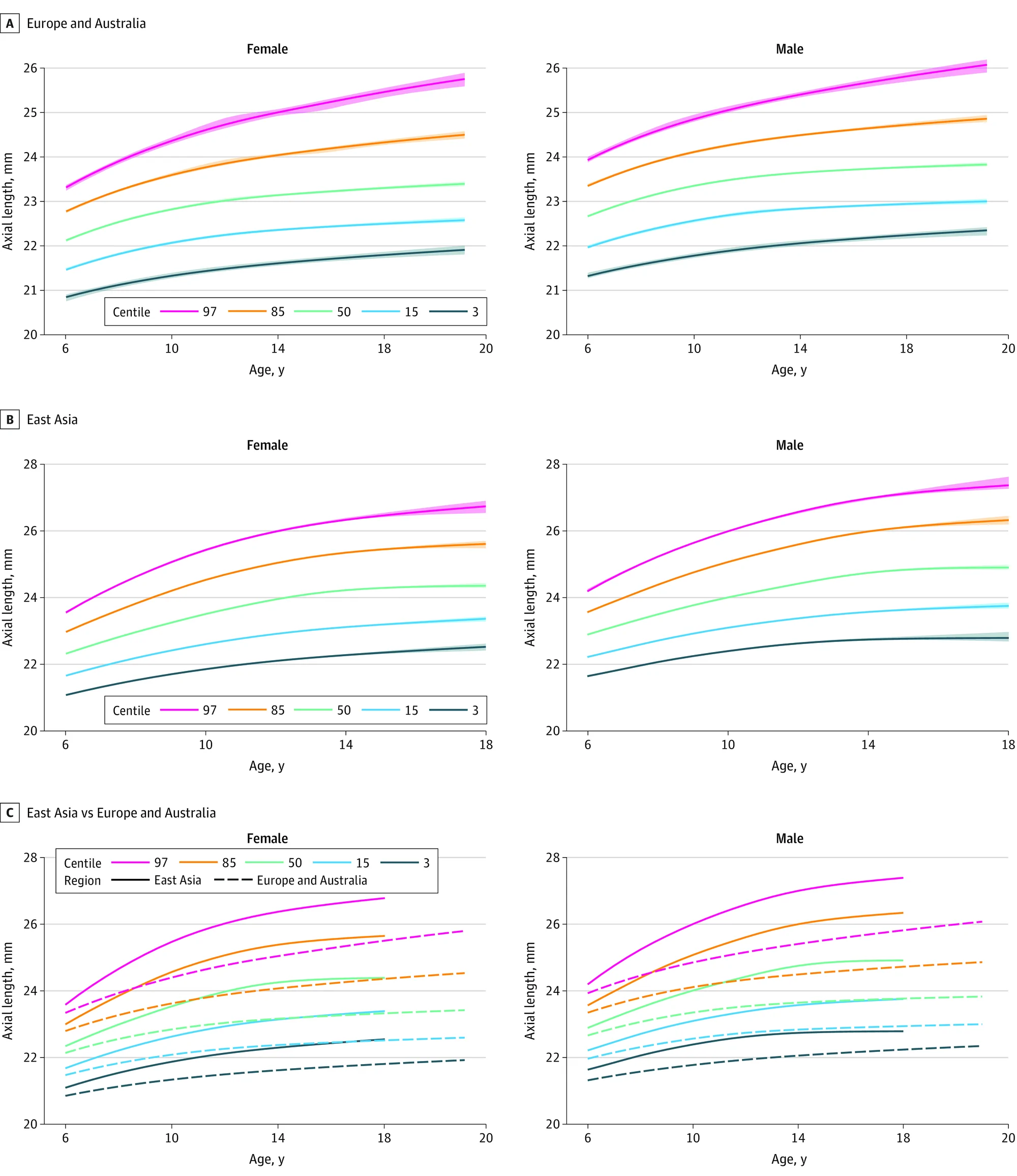
Centile Charts Showing Rates of Change in Axial Length

AL centiles and 95% CIs are presented in Figure 2, including the 3rd, 15th, 50th, 85th, and 97th centiles in line with the WHO methodology.^15^ The number of observations included for each region, sex and age subgroup, and additional percentile values are provided in eTables 2, 3 and 4 in Supplement 1. While confidence intervals were small, we noted greater confidence intervals for the 3rd and 97th centiles as well as at ages greater than 16 years, reflecting greater uncertainty in these centile values. Across the presented centiles, male ALs were longer than female ALs by an average of 0.54 mm for the East Asian model and 0.46 mm for the Australian and European model. At every centile level, ALs were greater in the East Asian population compared with European and Australian populations (eFigure 3 in Supplement 1). These differences were more pronounced at higher centiles and increased with age (in female individuals, the 50th centile AL at age 7 years was 22.35 mm in Europe and Australia and 22.67 mm in East Asia; by age 18 years, the 50th centile ALs were 23.31 mm and 24.36 mm, respectively). For example, among female individuals at age 7 years, ALs in the East Asian cohorts were higher than European and Australian values by 0.33 mm, 0.31 mm, and 0.50 mm at the 3rd, 50th, and 97th centiles, respectively. By age 18 years, the corresponding differences had widened to 0.73 mm, 1.05 mm, and 1.27 mm, respectively. A comparison of the current AL centiles with those from previously published studies is shown in eFigure 4 in Supplement 1. Overall, our new centiles demonstrated good agreement with the existing curves, particularly for the more central centiles.^19,20,21,22^

**Figure 3.**
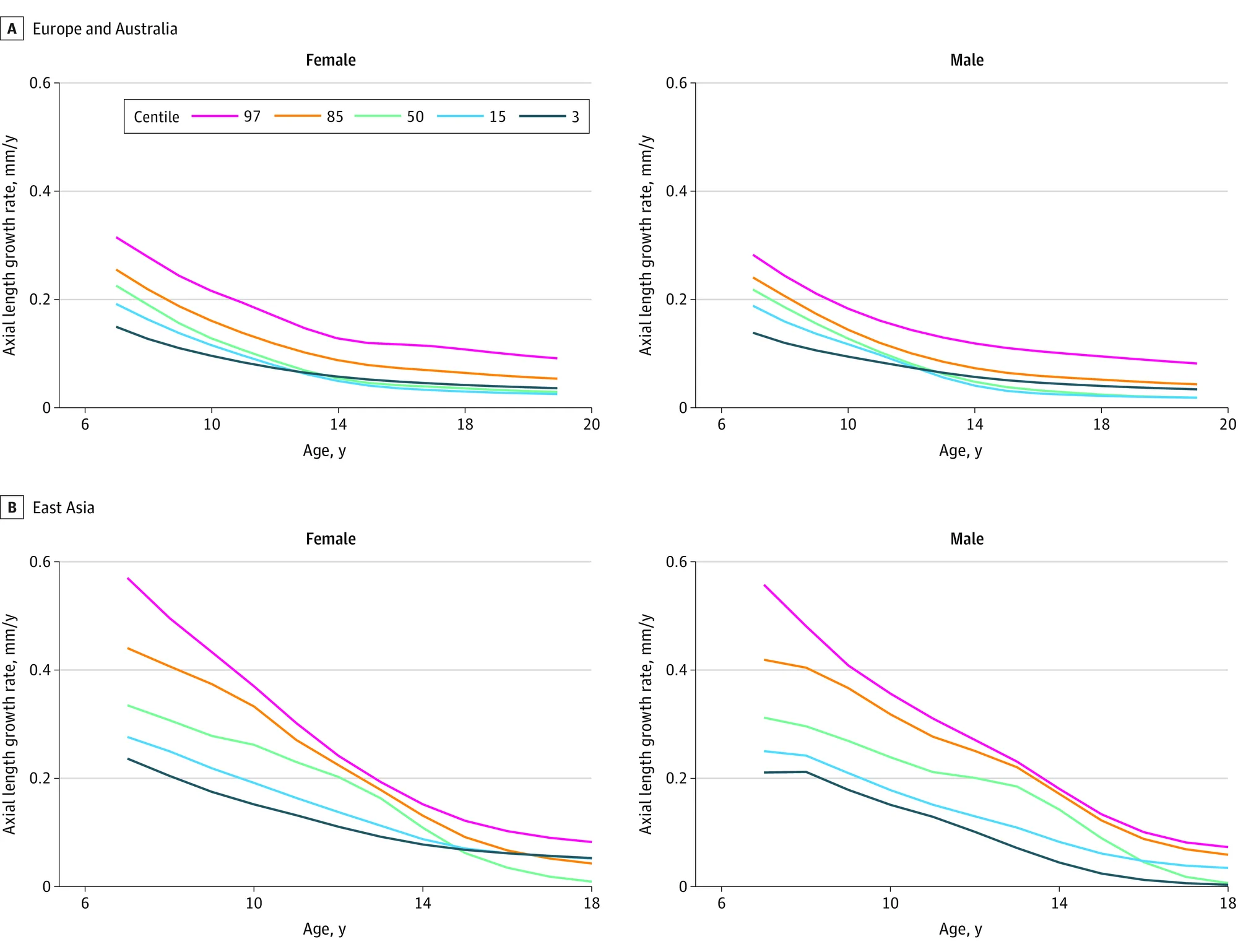
Bland-Altman Plots Comparing the Model Axial Length Centile Values With the Empirical Axial Length Centile Values Empirical estimates were derived by pooling all available data (Shanghai Time Outside to Reduce Myopia [STORM] data and 43.1% of data from the Generation R study were not included due to data-sharing constraints) and calculating axial length quantiles within pooled sex and region subgroups. Each point represents the difference between the model centile (eg, 15th centile at age 8 years, male, East Asia is 22.71 mm) and the corresponding empirical quantile (eg, 22.73 mm); this information is shown for the 1%, 2%, 3%, 5%, 10%, 15%, 25%, 50%, 75%, 85%, 90%, 95%, 97%, 98%, and 99% centiles. The solid line indicates the mean bias, the dashed lines the 95% limits of agreement, and the dotted lines the 95% CIs for the limit of agreement. Across ages 6, 9, 12, 15, and 18 years, the mean bias ±95% limits of agreement were 0.04 mm ±0.28 for European and Australian female individuals, 0.02 mm ±0.20 for European and Australian male individuals, −0.08 mm ±0.24 for East Asian female individuals, and −0.06 mm ±0.16 for East Asian male individuals.

The rate of axial growth, as inferred from change in AL centile values, is illustrated in Figure 3, with corresponding values provided in eTable 5 in Supplement 1. Despite differences in absolute AL between sexes, axial growth rates appeared similar between male and female participants. Regional differences were most apparent between ages 7 and 13 years, during which age range East Asian children exhibited faster growth across centiles. For example, growth at the 50th centile was 0.24 mm/year in East Asian boys vs 0.13 mm/year in European and Australian boys. By age 14 to 18 years, growth slowed in both regions and regional differences narrowed or reversed; for instance, at the 50th centile, growth was 0.05 mm/year in East Asian boys vs 0.03 mm/year in European/Australian boys. Comparable patterns were observed among girls (eTable 5 in Supplement 1).

## Discussion

In this study, large, pooled cohorts from East Asia, Europe, and Australia showed marked intercontinental and sex differences in AL growth from childhood to adolescence. These new centile charts provide reference standards that might be used for myopia research.

Using the dataset from the CREAM-Kids Consortium, we developed region- and sex-specific AL centile charts for East Asian children (aged 6-18 years) and European/Australian children (aged 6-21 years). Differences between European and Australian cohorts were small (approximately 1% of total variance), supporting their combination. Across all plotted centiles and ages, male participants had longer ALs than female participants in both the East Asian (Δ = 0.54 mm) and European and Australian (Δ = 0.46 mm) groups, supporting the general rule of thumb that the sex difference in eye size is approximately 0.5 mm.

East Asian children consistently showed longer AL across the entire centile range, with differences increasing with age, particularly in the upper centiles. The East Asian median curve approximated the 85th percentile of the European and Australian curve, with divergence widening until approximately 14 years and stabilizing thereafter. Between ages 7 and 13 years, East Asian children showed faster axial elongation than European and Australian children across the centile range, consistent with earlier and more rapid myopia development in East Asian populations.^1^ By mid to late adolescence, growth rates slowed in both regions and regional differences narrowed or reversed. These observations reflect differences in the centile trajectories rather than results of statistical testing, as formal inference on growth-rate differences is not feasible within the current analysis. A similar pattern was observed among female participants. This narrowing and even reversal of regional differences at older ages is consistent with approximately one-third of Australian adults reportedly having myopia progression of −0.50 D or more after the age of 20 years and a marked increase in adult-onset myopia in the UK at these ages.^23,24^

Regional differences may reflect environmental influences, including less time outdoors, greater near work, and higher educational demands, which often occur earlier in East Asian settings.^25,26,27^ Previous studies have shown that East Asian children spend more time on near work and less time outdoors compared with their European and Australian peers.^28^ Children of East Asian ancestry growing up in Sydney, Australia had significantly lower myopia prevalence and greater outdoor exposure than children of East Asian ancestry in Singapore, suggesting that environmental context may even outweigh ethnic background in determining myopia risk.^11^ Furthermore, genetic variants associated with myopia are shared across European and Asian ancestry groups.^29^

Our results align with prior studies^19,20,21,30^ showing longer AL in male individuals and consistently longer AL in East Asian cohorts. Despite these similarities, direct comparison with earlier growth curves remains challenging as varying percentile definitions and statistical approaches to model growth trajectories were used. To make comparisons possible, we generated AL values for corresponding centiles and ages reported in the literature^19,20,21,22^ (eFigure 4 in Supplement 1). Our European and Australian AL centile curves show good agreement with previously published curves,^19,20,21,30,31^ particularly in the central centiles (eg, 25th to 75th). In contrast, the East Asian curves from He et al^20^ display a noticeably steeper increase in AL after age 15 than our model, while the curves from Sanz Diez et al^31^ differ more substantially across the age range. This may reflect differences in the timing of data collection across cohorts, as well as variation in myopia prevalence between study populations. The data from He et al were included in the CREAM-Kids dataset; however, their steeper growth trajectory was moderated in the final model by the addition of other East Asian cohorts. The greater divergence from the curves presented in the study by Sanz-Diez et al likely reflects that their centiles are derived from a single-city cohort, whereas our model pools multiple large cohorts across East Asia. The resulting flattening of the combined curve may therefore reflect a combination of regional lifestyle differences, sampling variation, and heterogeneity in cohort composition.

### Limitations

Some limitations of the study should be noted. First, we have created region-specific eye growth percentile curves, rather than growth standards; thus, our curves do not represent healthy growth. As described by the WHO, ideal growth standards should be based on high-quality longitudinal data with 1-year follow-up intervals, drawn from healthy—and in our case, emmetropic—populations, and measured using standardized protocols.^12^ Thus, eye growth standards should be drawn from longitudinal studies specifically designed for this purpose with closer sampling and standardized measurement techniques.^15^ It may be that the differences noted in this study are driven by myopia and thus will be absent in an analysis of emmetropic participants. Therefore, the AL curves generated in the present study should not be interpreted as defining healthy or unhealthy eye growth. Rather, they serve to compare a child’s growth with that of peers. We plan to develop growth curves stratified by refractive category, and to compare individuals who develop myopia with those who remain emmetropic. Another limitation is that although AL provides an overall indicator of ocular elongation, it does not capture localized changes such as posterior staphylomas or other structural deformities. These features are rare in individuals younger than 25 years, but their presence could still influence interpretation of AL measurements, particularly at higher ALs.^32^

Also, we did not correct for participant height or year of data collection, both of which can influence AL.^3,33^ Previous studies have reported that AL increases by 0.03 mm for every 1-cm increase in height^33^ and by 0.006 mm per calendar year.^3^ Based on these estimates, the expected difference in AL due to average adult male height differences between Europe (180 cm) and East Asia (174 cm) would be approximately 0.18 mm.^34^ In addition, the collection period across the consortium studies ranged from 1999 to 2024, a span of 25 years. Given the rate of 0.006 mm increase in AL per year, this corresponds to a maximum potential difference of 0.15 mm over 25 years (0.006 mm/y × 25 years).^3^ This suggests that the contributions of such factors are small and their overall effect on the modeled centiles is minimal. Additionally, the dataset includes both population-based and school-based cohorts, so regional AL distributions may be influenced by differences in participation patterns, recruitment settings, or underlying refractive status distributions, which could shift absolute percentile estimates while likely having minimal impact on the overall shape of growth trajectories. In addition, although the mixed-effects model accounts for study-level variability through the inclusion of random effects, potential differences between school-based and population-based study designs may still influence the findings.

## Conclusions

This pooled cohort study presents a set of region- and sex-specific AL centile charts for East Asia, Europe, and Australia, providing a reference framework for interpreting ocular growth during childhood and adolescence. The curves offer insight into key differences in growth trajectories, with East Asian children demonstrating faster axial elongation at younger ages compared with their European and Australian peers. Although these charts are not intended to diagnose myopia, the charts can support clinicians in interpreting AL measurements and estimating a child’s projected adult AL. As the charts are derived from both population-based and school-based cohorts, absolute percentile levels may reflect this mixed sampling. Future research might aim to develop refractive-error stratified references and further standardize ocular biometry protocols to enhance the precision and clinical applicability of growth modeling.

## Notes

Supplement 1. eAppendix 1. Supplemental cohort information eAppendix 2. Supplementary Results eAppendix 3. Supplementary discussion eFigure 1. Median axial length by region and sex with quantile regression fits (black lines, fit using age and logarithm of age as explanatory variables). eFigure 2. Comparison of Multi-gaussian vs Box-Cox Power Exponential (BCPE) vs a square root transformation and normal distribution fit eFigure 3. Comparison of axial length centile curves from East Asian cohorts (solid lines) and European and Australian cohorts (dashed lines) eFigure 4. Comparison of axial length centiles in the current study (CREAM-Kids) to published studies. eFigure 5. Sensitivity analysis comparing the model used in the current study (referred to as a “Two-step” model to a Generalised Additive Model of Location Scale and Shape (GAMLSS) centile model fit using the Box-Cox Power Exponential distribution. eFigure 6. Sensitivity analysis comparing the model used in the current study with data from all eyes from all visits with the same model using only data from one eye per visit across all visits. eFigure 7. Sensitivity analysis comparing the model used in the current study with data from all eyes from all visits with the same model using only data from one eye per person across all visits. eTable 1. Leave one out analysis assessing the influence of individual studies within-region variance estimates. eTable 2. Number of observations using either persons, visits or eyes as the unit of analysis eTable 3. Axial length centile values for European and Australian males and females across a selected range of centiles eTable 4. Axial length centile values for East Asian males and females eTable 5. Annualised rates of change in axial length centile values for males and female from East Asia or Europe and Australia

Supplement 2. CREAM-Kids Consortium members

Supplement 3. Data sharing statement
