# Supplementary material for: Global Axial Length Centile Charts
Source: JAMA Ophthalmol. 2026 Jul 16:e262539. Online ahead of print. doi: 10.1001/jamaophthalmol.2026.2539 (PMC13377471; doi:10.1001/jamaophthalmol.2026.2539)
Supplement: Supplement 1. — eAppendix 1. Supplemental cohort information eAppendix 2. Supplementary Results eAppendix 3. Supplementary discussion eFigure 1. Median axial length by region and sex with quantile regression fits (black lines, fit using age and logarithm of age as explanatory variables). eFigure 2. Comparison of Multi-gaussian vs Box-Cox Power Exponential (BCPE) vs a square root transformation and normal distribution fit eFigure 3. Comparison of axial length centile curves from East Asian cohorts (solid lines) and European and Australian cohorts (dashed lines) eFigure 4. Comparison of axial length centiles in the current study (CREAM-Kids) to published studies. eFigure 5. Sensitivity analysis comparing the model used in the current study (referred to as a “Two-step” model to a Generalised Additive Model of Location Scale and Shape (GAMLSS) centile model fit using the Box-Cox Power Exponential distribution. eFigure 6. Sensitivity analysis comparing the model used in the current study with data from all eyes from all visits with the same model using only data from one eye per visit across all visits. eFigure 7. Sensitivity analysis comparing the model used in the current study with data from all eyes from all visits with the same model using only data from one eye per person across all visits. eTable 1. Leave one out analysis assessing the influence of individual studies within-region variance estimates. eTable 2. Number of observations using either persons, visits or eyes as the unit of analysis eTable 3. Axial length centile values for European and Australian males and females across a selected range of centiles eTable 4. Axial length centile values for East Asian males and females eTable 5. Annualised rates of change in axial length centile values for males and female from East Asia or Europe and Australia [file jamaophthalmol-e262539-s001.pdf]

Supplemental Online Content

Kneepkens SCM, Lingham G, van Hemert DJ, et al; CREAM-Kids Consortium. Global axial length centile charts. *JAMA Ophthalmol*. Published online July 16, 2026. doi:10.1001/jamaophthalmol.2026.2539

eMethods. Supplemental cohort information

eResults

eDiscussion

eFigure 1. Median axial length by region and sex with quantile regression fits (black lines, fit using age and logarithm of age as explanatory variables).

eFigure 2. Comparison of Multi-gaussian vs Box-Cox Power Exponential (BCPE) vs a square root transformation and normal distribution fit

eFigure 3. Comparison of axial length centile curves from East Asian cohorts (solid lines) and European and Australian cohorts (dashed lines)

eFigure 4. Comparison of axial length centiles in the current study (CREAM-Kids) to published studies.

eFigure 5. Sensitivity analysis comparing the model used in the current study (referred to as a “Two-step” model to a Generalised Additive Model of Location Scale and Shape (GAMLSS) centile model fit using the Box-Cox Power Exponential distribution.

eFigure 6. Sensitivity analysis comparing the model used in the current study with data from all eyes from all visits with the same model using only data from one eye per visit across all visits.

eFigure 7. Sensitivity analysis comparing the model used in the current study with data from all eyes from all visits with the same model using only data from one eye per person across all visits.

eTable 1. Leave one out analysis assessing the influence of individual studies within-region variance estimates.

eTable 2. Number of observations using either persons, visits or eyes as the unit of analysis

eTable 3. Axial length centile values for European and Australian males and females across a selected range of centiles

eTable 4. Axial length centile values for East Asian males and females

eTable 5. Annualised rates of change in axial length centile values for males and female from East Asia or Europe and Australia

This supplemental material has been provided by the authors to give readers additional information about their work.

© 2026 Kneepkens SCM et al. *JAMA Ophthalmology*.

eMethods. Supplemental cohort information

1.1 The generation R study

The Generation R Study is a population-based prospective cohort study designed to examine environmental and genetic factors influencing health, growth, and development from fetal life through young adulthood. Participants were recruited in Rotterdam the Netherlands. The cohort includes 9,778 mothers with delivery dates between April 2002 and January 2006, with a baseline response rate of approximately 61%. Follow-up rates have consistently remained around 80% until the age of 18 years. Data collection involves detailed physical examinations, biological sampling, and questionnaires for children and their parents. Eye-specific assessments performed in children include axial length measurements using optical biometry (IOLMaster 700), cycloplegic autorefraction, visual acuity testing, MRI images, and retinal imaging (fundus photography and Optical Coherence Tomography). These ophthalmological examinations were conducted periodically between ages 6 and 21 years. Findings from Generation R contribute to understanding ocular development and identifying potential preventive strategies for childhood eye conditions, particularly myopia. The complete methodology for the Generation R study has been described elsewhere. [1, 2] The Generation R study protocol was approved by the Medical Ethical Committee of the Erasmus Medical Centre, Rotterdam (MEC 217.595/2002/20). All participants provided written informed consent following the Declaration of Helsinki to participate in the study and consent for data collection from their treating physicians. The Generation R study is made possible by financial support from the Erasmus Medical Center, Rotterdam, The Netherlands; the Netherlands Organization for Scientific Research (NWO); the Netherlands Organization for Health Research and Development (ZonMw); the Dutch Ministry of Education, Culture and Science; the Dutch Ministry of Health, Welfare, and Sports; and the European Commission (DG XII).

1.2 Avon Longitudinal Study of Parents and Children (ALSPAC)

The ALSPAC Study is a population-based prospective cohort study designed to examine environmental and genetic factors influencing health and well-being of children and parents living in the United Kingdom (UK). Pregnant women resident in Avon, UK with expected dates of delivery between 1st April 1991 and 31st December 1992 were invited to take part in the study. 20,248 pregnancies have been identified as being eligible and the initial number of pregnancies enrolled was 14,541. The initial pregnancies resulted in 14,062 live births and 13,988 children who were alive at 1 year of age. When the oldest children were approximately 7 years of age, an attempt was made to bolster the initial sample with eligible cases who had failed to join the study originally. As a result, the total sample size for the current analyses was 15,447 pregnancies, resulting in 15,658 foetuses, of whom 14,901 children were alive at 1 year of age. Data collection has involved detailed physical examinations, biological sampling, and questionnaires for children and their.[3, 4] Eye-specific assessments include visual acuity, stereopsis, non-cycloplegic autorefraction, axial length measurement and keratometry. Ethical approval for the study was obtained from the ALSPAC Ethics and Law Committee and the Local Research Ethics Committees (Refs: E1808/E4168/E5215/E5691/E5806/06/Q2006/53). Informed consent for the use of data collected via questionnaires and clinics was obtained from participants following the recommendations of the ALSPAC Ethics and Law Committee at the time. The ALSPAC study website contains details of all the data that is available through a fully searchable data

© 2026 Kneepkens SCM et al. *JAMA Ophthalmology*.

dictionary and variable search tool" and reference the following webpage: http://www.bristol.ac.uk/alspac/researchers/our-data/

1.3 CISViT Project

The Terrassa Visual Health Children's Cohort (CISViT – Cohort Infantil de Salut Visual de Terrassa) is a pioneering schoolchildren cohort study in the Southern European population which involves 16 schools in the city of Terrassa (Barcelona, Spain). This prospective longitudinal epidemiological cohort study aims to study changes in visual function and ocular parameters in children from 8 to 16 years of age.

The initial data collection phase involves a vision screening at the Centre Universitari de la Visio (University Vision Center; the Universitat Politècnica de Catalunya optometry clinic) comprising monocular measurements of distance visual acuity using Snellen charts, non-cycloplegic objective autorefraction and retinoscopy and ocular biometric measurements, including axial length (AL) and corneal curvature radius (CR), by AL-SCAN M biometer (Nidek Co., Japan). Additionally, a distance and near cover test were conducted to detect binocular vision misalignments.

In the follow-up phases a team of optometrists attend the schools to provide the vision screening within the school premises.

In addition to the tests conducted, prior to each vision screening, participants' parents are required to complete a detailed questionnaire, which collect information of ethnic ancestry, myopic inheritance, socioeconomic factors, and lifestyle.

This study was approved by the Drugs Medication Ethics Committee (CEIm) of Mutua Terrassa (P/22-090) and designed in accordance with the Declaration of Helsinki. Written informed consent is obtained from each parent or guardian prior to the involvement in the study, ensuring that they fully understand the nature and purpose of the research. Informed consent is also required and re-confirmed at the follow-up visits.

1.4 Glasgow Caledonian Study

The Glasgow Caledonian Study is a population-based longitudinal study of refractive development in children and young adults. The aim of this study was the characterisation of refractive error progression and the identification of patterns influencing structural, functional, genetic and environmental factors in the development of refractive error. Participants were recruited while attending for regular eye examinations at a clinical optometry practice in Duisburg, Germany in 2006. The cohort included 140 participants (35 myopes, 61 emmetropes and 44 hyperopes on initial visit) were aged between 5 and 20 years on the initial visit. Data collection involves detailed questionnaires for children and their parents relating to lifestyle and genetic information including indoor and outdoor activities, reading habits and sporting achievement. Eye-specific assessments performed included axial length and anterior chamber depth measurements using optical biometry (IOLMaster 700), non-cycloplegic autorefraction, keratometry, visual acuity testing, peripheral refraction and oculomotor assessment. The data were collected at baseline and 2 and 4 years after the initial visit. Other data collected included body height at each visit. The Glasgow Caledonian study protocol was approved by the Health and Life Sciences Ethical Committee of Glasgow Caledonian University ("An Investigation of the Nature of Refractive Development in Children and Young Adults" 2006). All participants provided written informed consent for data collection from the research optometrist.

© 2026 Kneepkens SCM et al. *JAMA Ophthalmology*.

1.5 Ireland Eye Study

The Ireland Eye Study, a cross-sectional, population-based epidemiological study, was designed to assess the prevalence of refractive error and visual function in school-aged children across Ireland. Conducted between 2016 and 2017, the study recruited 1,626 children aged 6–7 and 12–13 years using stratified random cluster sampling, with schools serving as clusters. Sampling was stratified by school type (primary/post-primary), socioeconomic status (DEIS/non-DEIS), and urban/rural location, ensuring national representativeness. Examinations were conducted on school premises and included presenting (with spectacles if worn), unaided, and pinhole LogMAR visual acuity, stereoacuity, ocular motility, colour vision, cycloplegic autorefraction (using 1% cyclopentolate), ocular biometry (IOLMaster 500), height and weight, ocular dominance, and hand laterality. Comprehensive parental questionnaires gathered demographic and lifestyle data. The IES adhered to international protocols for cycloplegic refraction and aimed to provide reliable, standardised data for comparison with other global paediatric eye health studies. The study was approved by the TU Dublin Research Ethics Committee and conducted in accordance with the Declaration of Helsinki, with informed consent obtained from parents/legal guardians and assent from participating children.

1.6 Finnish conscripts study

In the summer of 2022, a total of 1862 conscripts commenced military service at Vekarajärvi garrisons. Of these, 1694 individuals (mean age of 19.3 ±1.55 years) participated voluntarily in this study at the onset of their service. During the initial days, participants completed a structured questionnaire and data collection, including autorefraction. Men whose home language was Finnish and who accepted tropicamide cycloplegia were included in the study. Cycloplegia was induced using tropicamide eye drops (Oftan Tropicamid 5mg/ml ®, Santen, Japan) with one drop administered to each eye. Approximately 30 minutes post-administration, refractive measures were conducted using an autorefractometer (NIDEK AR-1s®, Tokyo, Japan). The structured questionnaire collected demographic and behavioral data, including age, sex, home language, and estimated daily duration (in hours) of near work, outdoors, and smartphone usage These durations were recorded with an accuracy of one hour (categories: ≤ 1, 2, 3, 4, 5, 6, 7, or ≥8 hours). Educational attainment was classified into four categories based on the highest educational level achieved: obligatory basic school (starting at the age of seven, lasting for nine years), vocational school and upper secondary school (both lasting for three years after basic school). And the fourth education group (highly educated) comprised of those who had continued their education either to a university (N =135) or to a university of applied sciences (n=50). No statistically significant difference in myopia prevalence was observed between the university students (31.1%) and the university of applied sciences students (28.0%) In nine cases, refractive surgery was performed. Their preoperative refraction values were obtained from the clinics where the surgeries were performed. Biomertic measures were done (about half and half= with Haag-Streit AG. Lenstar LS 900 APS and Carl Zeiss Meditec AG. IOLMaster 500)

1.7 The Northern Ireland Childhood Errors of Refraction (NICER) study

The Northern Ireland Childhood Errors of Refraction (NICER) study is a prospective population-based cohort study designed to investigate the prevalence, progression and determinants of refractive error among children and young adults in Northern Ireland. The study used stratified, random cluster sampling to recruit a representative sample of children

© 2026 Kneepkens SCM et al. *JAMA Ophthalmology*.

aged 6–7 years (younger cohort) and 12–13 years (older cohort) between 2006 and 2008 (baseline) from schools in Northern Ireland. The sampling method ensured representation from both rural/urban and deprived/non-deprived areas of Northern Ireland. A total of 1,047 white children were enrolled at baseline including 390 of these were aged 6–7 years and 657 aged 12 – 13 years with an initial response rate of approximately 60%. Participants were re-assessed prospectively at three, six and nine years from baseline. Follow up data were available for at least one visit from 323 (83%) participants within the younger cohort and 480 (73%) of the older cohort. Data collection included cycloplegic autorefraction (Shin-Nippon SRW-5000 or Nvision-k 5001), ocular biometry (IOLMaster v3), distance visual acuity, assessment of heterophoria/tropia, anthropometric measurements, fundus photography and lifestyle questionnaires. The NICER study provides, robust, UK-specific epidemiological data on the prevalence of myopia[5], hyperopia[5] and astigmatism[6] and has been instrumental in identifying environmental and demographic risk factors associated with myopia development and progression.[6-8] The complete methodology for the NICER study is published elsewhere.[9] The study was approved by Ulster University's Research Ethics committee (REC/18/0102) and adhered to the tenets of the Declaration of Helsinki. Written informed consent was obtained from parents or guardians and verbal or written assent was obtained from participants on the day of the examinations at each study visit. The study was funded by The College of Optometrists, UK and Ulster University.

1.8 Refractive Error and Color Vision Study (Norwegian 16–19-year-olds)

As part of the Refractive Error and Color Vision Study conducted in Norway from 2014 to 2020, a cohort of 16–19-year-olds was measured twice over a two-year period. The study was observational with an aim to examine associations between refractive errors, color vision, cone opsin genetics, cone ratios, and behavior in adolescents and young adults. Participants were recruited in South-Eastern Norway. The study included 439 participants (184 males and 255 females), who were first examined in 2015–2016. A sample of 93 participants (59 females and 34 males) were measured a second time in 2018. Data collection involved questionnaires as well as blood and saliva samples. Eye-specific assessments included ocular biometry measurements (IOLMaster 500 or 700), cycloplegic autorefraction, visual acuity testing, and color vision testing. The methodology for these parts of the Refractive Error and Color Vision Study has been described elsewhere.[10, 11] The Refractive Error and Color Vision Study protocol was approved by the Regional Committees for Medical Research Ethics in South East Norway (Ref. No. 2014/1778). All participants provided written informed consent following the Declaration of Helsinki to participate in the study.

1.9 The Southeast Norway Vision and Visuomotor Study (SNOW)

The SNOW Study is a school-based prospective cohort study designed to examine development of refractive errors, vision and motor skills, and how these interact with health and learning in children and adolescents aged 7 to 16 years. Eye-specific assessments included ocular biometry (IOLMaster 700), cycloplegic and non-cycloplegic autorefraction, visual acuity and color vision testing. Additionally, questionnaires and motor skills testing were conducted.

The study started in autumn 2015, and was initiated by the National Centre for Optics, Vision and Eye Care at the University of South-Eastern Norway, as part of a long-running school vision program. This program is offered to all children in 2nd (7–8 years), 5th (10–11 years)

© 2026 Kneepkens SCM et al. *JAMA Ophthalmology*.

and 10th (15–16 years) grade in all community schools of the Kongsberg municipality in Norway. The SNOW Study collects additional measurements in four of these community schools for research purposes and to evaluate the quality of the school vision program. The study is ongoing, and new children are enrolled every year. Children enrolled in 2nd grade will be measured again in 5th and 10th grade, whereas children enrolled for the first time in 10th grade will only be measured once. The study will perform cross-sectional analyses to assess age, cohort, or period effects, alongside longitudinal analyses to track changes over time. As of March 2025, the cohort included 1945 children of whom 559 have been measured twice and 62 three times (as they have passed through 2nd, 5th and 10th grades). The first data from the study on refractive errors and ocular biometry and the methodology is described in a paper that is currently under review in a journal. The SNOW Study protocol was approved by the Regional Committees for Medical Research Ethics in South East Norway, first as part of the Refractive Error and Color Vision Study (Ref. No. 2014/1778), then with an updated protocol: The Southeast Norway Vision and Visuomotor Study (Ref. No. 2019/578) and Norwegian Agency for Shared Services in Education and Research for research (Ref. No. 618194) and quality assurance (Ref. No. 192931). Verbal information about the study, including possible consequences, was given to parents/guardians attending parents' evenings organized by the schools, before informed written consent was obtained from both parents/guardians. Verbal consent was also obtained from the children themselves before any procedure was started. Adolescents aged 16 years or older provided informed written consent, as they are considered fully competent to consent to participate in research according to the Norwegian Health Research Act.

1.10 The Singapore Cohort of Risk factors for Myopia (SCORM)

Singapore Cohort Study of the Risk Factors of Myopia (SCORM) is a prospective cohort where children from grades 1 to 3, aged 7 to 9 years, were recruited from three Singapore schools (n = 1979), with the methodology described previously.[12, 13] At baseline, children were excluded if they had serious medical conditions or any eye disorders, such as congenital cataract. Questionnaires were administered to obtain demographic data, including nearwork, outdoor and the number of parents with myopia. [12] Cycloplegic refraction using the autorefractor was performed at every visit with 3 drops of 1% cycloplentolate. Axial length and other biometry data were obtained using the A-scan biometry machine. The first four annual follow-up visits at the schools were included in this study.

1.11 West China Refractive Error Development Project (WCRES)

The WCRES is an ongoing population-based repeated cross-sectional study. This project started in the Fall of 2019, under the Chinese government's initiative on "National Screening and Intervention of Common Diseases and Health Risk Factors in Students 2019". Each year, students from 88 schools (including primary, middle, and high schools) in Qingyang District, Chengdu, China undergoing myopia screening are included in this project. The project collects data from myopia screening, which includes slit lamp examinations, visual acuity test using tumbling E visual charts, non-cycloplegic refractive error measurement using NIDEK auto-refractor (ARK-510A; NIDEK Corp., Tokyo, Japan), and axial length measurement using AL-Scan (NIDEK Corp., Tokyo, Japan). Detail of the WCRES was previous described elsewhere.[14] All data collection and eye examinations followed the tenets of the Declaration

© 2026 Kneepkens SCM et al. *JAMA Ophthalmology*.

of Helsinki. The nature of the study was explained to the participating children and their parents through the school, and verbal informed consent was obtained from parents before the commencement of the study.

1.12 Sydney Myopia Study

The Sydney Myopia Study is a population-based random cluster-sample of children in two age cohorts, 6 years (n=1765) and 12 years (n=2353) from 55 schools across the Sydney metropolitan area. Data collection was conducted from 2004 to 2005. The study area was divided into ten socioeconomic strata based on the Australian Bureau of Statistics (ABS) census. Two secondary and five primary schools were selected from the top strata, with the remaining randomly selected from the bottom nine strata, to ensure a representative sample. A proportional mix of public, academically selective and private/ religious schools were included. Detailed methodology for the Sydney Myopia Study has been previously reported.[15] All children underwent a comprehensive eye examination that included cycloplegic (Cyclopentolate 1%) autorefraction (Canon RK-F1, Tokyo, Japan) and ocular biometry (IOLMaster, Carl Zeiss, Meditec AG Jena, Germany). Ethnicity was determined by the self-identified ethnicity of both parents according to the Australian Standard Classification of Cultural and Ethnic Groups.[16] A detailed questionnaire provided previous medical and ocular history and estimates of time spent in a variety of activities.

Children who participated in the Sydney Myopia Study were prospectively followed up 5-6 years later (2009 to 2011) as part of the Sydney Adolescent and Vascular Eye Study. A total of 2103 children were re-examined, 892 (50.5%) from the younger cohort, then aged 12 years, and 1211 (51.5%) from the older cohort, then aged 17 years. Children in the same year group who had not participated in the baseline study were also invited to participate in the follow up, resulting in the addition of 475 children.

Both the Sydney Myopia Study and the Sydney Adolescent Vascular and Eye Study were approved by the Human Research Ethics Committee of the University of Sydney, the New South Wales Department of Education and Training and the Catholic Education Office. Informed written consent was obtained from parents or participants who were over the age of 18 years at the time of examination. Verbal consent was additionally obtained from all children prior to examination. The study adhered to the tenets of the Declaration of Helsinki.

1.13 Raine Study, Gen2

The Raine Study is a multi-generational, prospective, cohort study of pregnancy, childhood, adolescence and adulthood. From 1989 to 1991, 2,900 pregnant women were recruited at 16-18 weeks gestation were recruited to a randomised control trial of intensive ultrasound and Doppler studies on pregnancy outcomes.[17] From these pregnant mothers, 2,868 children were born, and these children formed the Raine Study Generation 2 (Gen2) cohort. The Gen2 cohort has been regularly followed-up since birth, including through childhood and adolescence.[18] Between 2010 and 2012, Gen2 Raine Study participants were invited to participate in a comprehensive eye examination as part of the Gen2-20 (20-year) follow-up. The number of active Raine Study Gen2 participants has gradually declined over time, with 2,135 active Gen2 participants at the time of the 20-year follow-up. Of these 1344 (77.1%) were examined as part of the eye health study of the 20- year follow-up.

At the Gen2-20 follow-up, eye specific assessments performed included visual acuity, autorefraction (before and after administration with Tropicamide 1% eye drops), ocular

© 2026 Kneepkens SCM et al. *JAMA Ophthalmology*.

biometry including axial length measurement (IOLmaster 500, Zeiss), intraocular pressure measurement, retinal imaging (fundus photography and Optical Coherence Tomography), anterior segment photography (colour and conjunctival auto-fluorescence imaging) and anterior segment tomography. Data was also collected on other variables including anthropomorphic measurements, socioeconomic and demographic data.[19, 20] All follow-ups of the Raine Study were conducted in accordance with the Declaration of Helsinki and were approved by the University of Western Australia Human Research Ethics Committee. Participants provided informed consent prior to participating in each follow-up.

1.14 Hong Kong Children Eye Study

The Hong Kong Children Eye Study (HKCES) is an on-going population-based cohort study, started from March 2015, designed to assess environmental and genetic factors associated with pediatric ocular diseases, particularly myopia, among school aged children. Participants recruit across Hong Kong, using a stratified random cluster sampling method from all primary schools registered under the Education Bureau. Till now, the cohort consists of more than 45,000 age 4 to 12 children and their parents. Data collection includes comprehensive eye examinations, basic physical assessments, and validated environmental and behavioral questionnaires. Eye-specific assessments included cycloplegic and non-cycloplegic autorefraction, axial length and corneal curvature via IOL Master 700, optical coherence tomography (SS-OCT/SD-OCT), fundus photography, intraocular pressure and corneal biomechanics (Corvis ST), corneal tomography, stereoacuity, color vision, and tests for binocular function and ocular movement. Findings from HKCES are expected to advance understanding of ocular development and myopia progression, particularly in high-prevalence populations like Hong Kong, and will inform early preventive interventions for myopia and other pediatric ocular conditions. A detailed methodology of HKCES has been described elsewhere.[21] The HKCES protocol was reviewed and approved by the Joint Chinese University of Hong Kong-New Territories East Cluster Clinical Research Ethic Committee (CREC Ref. No.: 2015.033), in accordance with the Declaration of Helsinki. All participants provided written informed consent, with parents or guardians consenting on behalf of the children.

1.15 GUSTO

The Growing Up in Singapore Towards healthy Outcomes (GUSTO) study is a prospective birth cohort designed to investigate how prenatal and early-life exposures influence long-term health and developmental outcomes in women and children. Between June 2009 and October 2010, 1450 pregnant women (Singapore citizens or permanent residents) at 7–11 weeks' gestation were recruited. Of these, 1209 women completed delivery (55.9% Chinese, 26.1% Malay, 18.0% Indian). Full methodological details were previously described[22-25]. In brief, participants were followed at gestational weeks 11-12, 19-21, 26-28, and 32-34. Birth tissues were obtained at delivery, and newborn anthropometry was conducted within 24 hours. Postnatal follow-up occurred at Week 3, every 3 months until 18 months, at 24 months, and then annually. GUSTO is currently in its 14th year. Ocular assessments were performed at year 3, year 6 and year 9 visits. At year 3 and 9, questionnaires were administered to capture data (e.g., type and duration) on near work and outdoor activities. Ocular measurements include unaided distance visual acuity, cycloplegic autorefraction and axial length. Cycloplegia at year 3 and year 6 involved one drop of 0.5% proparacaine,

© 2026 Kneepkens SCM et al. *JAMA Ophthalmology*.

followed by one drop of 2.5% phenylephrine, and then three drops of 1% cyclopentolate instilled at 5 min intervals. At Year 9, three drops of 1% cyclopentolate were instilled at 5 min intervals. Autorefraction was performed at least 30 minutes after the first drop, with pupil dilation of ≥ 6 mm. Myopia was defined as SE of ≤ -0.5 D. Axial length was measured using an optical biometer (IOLMaster; Carl Zeiss Meditec, Jena, Germany).

1.16 Shanghai Axial Length Data

The axial length data from Shanghai, China have examined participant data on axial length, cycloplegic spherical equivalent, and corneal curvature from 14,127 individuals aged 4 to 18 years, sourced from baseline data of three studies. Details of the design and methodology of two of these studies have been published previously.[26, 27] The third study included data from 1,817 young adults, primarily aged 17 to 18 years, from the Shanghai region. The combined data have been analyzed and the results have been published.[28] Informed consent was obtained from parents or guardians for all participants, and from the participants themselves in the case of young adults. The datasets excluded participants with any systemic or ocular pathologies, strabismus, amblyopia, or those who had undergone myopia control treatments. Each participant in the development dataset underwent a comprehensive eye examination, which included objective cycloplegic refraction using two drops of 1% cyclopentolate, preceded by 0.5% proparacaine. Refractive error was measured with an autorefractor (KR-8900, Topcon, Tokyo, Japan), while axial length was measured using an IOL Master (V.5.02, Carl Zeiss, Jena, Germany). Only baseline measurements were included in the analysis. Corneal curvature data, encompassing the radii of curvature for both steep and flat meridians, were also obtained from the IOL Master. Similarly, the validation dataset included cycloplegic refractive error assessments conducted with the autorefractor (KR-8900, Topcon, Tokyo, Japan) and measurements of AL and corneal curvature using the IOL Master (version 5.02, Carl Zeiss, Jena, Germany), with cycloplegia induced using two drops of 1% cyclopentolate.

1. Jaddoe VW, van Duijn CM, Franco OH, et al. The Generation R Study: design and cohort update 2012. *Eur J Epidemiol* 2012;27:739-756.
2. Kooijman MN, Kruithof CJ, van Duijn CM, et al. The Generation R Study: design and cohort update 2017. *Eur J Epidemiol* 2016;31:1243-1264.
3. Boyd A, Golding J, Macleod J, et al. Cohort Profile: the 'children of the 90s'--the index offspring of the Avon Longitudinal Study of Parents and Children. *Int J Epidemiol* 2013;42:111-127.
4. Fraser A, Macdonald-Wallis C, Tilling K, et al. Cohort Profile: the Avon Longitudinal Study of Parents and Children: ALSPAC mothers cohort. *Int J Epidemiol* 2013;42:97-110.
5. O'Donoghue L, McClelland JF, Logan NS, Rudnicka AR, Owen CG, Saunders KJ. Refractive error and visual impairment in school children in Northern Ireland. *Br J Ophthalmol* 2010;94:1155-1159.
6. O'Donoghue L, Breslin KM, Saunders KJ. The Changing Profile of Astigmatism in Childhood: The NICER Study. *Invest Ophthalmol Vis Sci* 2015;56:2917-2925.
7. McCullough S, Adamson G, Breslin KMM, McClelland JF, Doyle L, Saunders KJ. Axial growth and refractive change in white European children and young adults: predictive factors for myopia. *Sci Rep* 2020;10:15189.
8. McCullough SJ, O'Donoghue L, Saunders KJ. Six Year Refractive Change among White Children and Young Adults: Evidence for Significant Increase in Myopia among White UK Children. *PLoS ONE* 2016;11:e0146332.

© 2026 Kneepkens SCM et al. *JAMA Ophthalmology*.

9. O'Donoghue L, Saunders KJ, McClelland JF, et al. Sampling and measurement methods for a study of childhood refractive error in a UK population. *Br J Ophthalmol* 2010;94:1150-1154.
10. Hagen LA, Gjelle JVB, Arnegard S, Pedersen HR, Gilson SJ, Baraas RC. Prevalence and Possible Factors of Myopia in Norwegian Adolescents. *Sci Rep* 2018;8:13479.
11. Hagen LA, Gilson SJ, Akram MN, Baraas RC. Emmetropia Is Maintained Despite Continued Eye Growth From 16 to 18 Years of Age. *Invest Ophthalmol Vis Sci* 2019;60:4178-4186.
12. Tong L, Saw S-M, Siak J-K, Gazzard G, Tan D. Corneal Thickness Determination and Correlates in Singaporean Schoolchildren. *Investigative Ophthalmology & Visual Science* 2004;45:4004-4009.
13. Saw SM, Tong L, Chua WH, et al. Incidence and progression of myopia in Singaporean school children. *Invest Ophthalmol Vis Sci* 2005;46:51-57.
14. Pan W, Lin J, Zheng L, et al. Myopia and axial length in school-aged children before, during, and after the COVID-19 lockdown-A population-based study. *Front Public Health* 2022;10:992784.
15. Ojaimi E, Rose KA, Smith W, Morgan IG, Martin FJ, Mitchell P. Methods for a population-based study of myopia and other eye conditions in school children: the Sydney Myopia Study. *Ophthalmic Epidemiol* 2005;12:59-69.
16. Statistics ABo. Australian Standard Classification of Cultural and Ethnic Groups (ASCCEG). ABS; 2019.
17. Newnham JP, Evans SF, Michael CA, Stanley FJ, Landau LI. Effects of frequent ultrasound during pregnancy: a randomised controlled trial. *Lancet* 1993;342:887-891.
18. Straker L, Mountain J, Jacques A, et al. Cohort Profile: The Western Australian Pregnancy Cohort (Raine) Study-Generation 2. *Int J Epidemiol* 2017;46:1384-1385j.
19. Yazar S, Forward H, McKnight CM, et al. Raine eye health study: design, methodology and baseline prevalence of ophthalmic disease in a birth-cohort study of young adults. *Ophthalmic Genet* 2013;34:199-208.
20. Lee SS, Lingham G, Yazar S, et al. Rationale and protocol for the 7- and 8-year longitudinal assessments of eye health in a cohort of young adults in the Raine Study. *BMJ Open* 2020;10:e033440.
21. Yam JC, Tang SM, Kam KW, et al. High prevalence of myopia in children and their parents in Hong Kong Chinese Population: the Hong Kong Children Eye Study. *Acta Ophthalmol* 2020;98:e639-e648.
22. Soh S-E, Tint MT, Gluckman PD, et al. Cohort profile: Growing Up in Singapore Towards healthy Outcomes (GUSTO) birth cohort study. *International journal of epidemiology* 2014;43:1401-1409.
23. Chua SYL, Ikram MK, Tan CS, et al. Relative contribution of risk factors for early-onset myopia in young Asian children. *Investigative ophthalmology & visual science* 2015;56:8101-8107.
24. Chen DZ, Wong C, Lam JSH, et al. Axial length elongation profiles from 3 to 6 years in an Asian paediatric population: the Growing Up in Singapore Towards Healthy Outcomes birth cohort study (GUSTO). *British Journal of Ophthalmology* 2024;108:1018-1023.
25. Li M, Lanca C, Tan C-S, et al. Association of time outdoors and patterns of light exposure with myopia in children. *British Journal of Ophthalmology* 2023;107:133-139.
26. Sankaridurg P, He X, Naduvilath T, et al. Comparison of noncycloplegic and cycloplegic autorefraction in categorizing refractive error data in children. *Acta Ophthalmol* 2017;95:e633-e640.
27. He X, Sankaridurg P, Xiong S, et al. Shanghai Time Outside to Reduce Myopia trial: design and baseline data. *Clin Exp Ophthalmol* 2019;47:171-178.
28. He X, Sankaridurg P, Naduvilath T, et al. Normative data and percentile curves for axial length and axial length/corneal curvature in Chinese children and adolescents aged 4-18 years. *Br J Ophthalmol* 2023;107:167-175.

© 2026 Kneepkens SCM et al. *JAMA Ophthalmology*.

© 2026 Kneepkens SCM et al. *JAMA Ophthalmology*.

eResults

Sensitivity analyses

Supplementary Figures 5, 6 and 7 compare the current centile model to centile models generating using i) GAMLSS ii) the current model using data from one randomly selected eye per visit and iii) the current model using data from one randomly selected eye per person (across all visits). Comparing the current model and GAMLSS, there was little difference in the 15th, 50th and 85th centiles, generally being with ±0.1mm across all age, sex and regions groups. Difference were larger differences for the 3rd and 97th centiles, being up to ±0.30mm. Comparing the current model using data from all eyes vs one eye per visit, there were minimal differences across all centiles (all within ±0.075mm), while comparing against one eye per person centile differences were within ±0.15mm, the largest differences being for older age groups where data were already sparser.

Evaluation of axial length centile models

To evaluate the internal consistency of the generated AL growth models, we compared the AL centile values from the model (Supplementary Table 2) to the empirical AL centile values (illustrated in Supplementary Figure 8) extracted from pooled European/Australian studies (43·1% of Generation R data not included) or pooled East Asian studies (STORM study not included). Empirical centile values were extracted by age and sex for each region. The modelled and empirical AL centiles showed good agreement, with intraclass correlations of >0·99 for all sex and region categories. Figure 3 presents Bland-Altman plots comparing modelled and empirical AL centile values. Both models showed a mean bias close to zero. For the East Asian data, a slight negative bias was observed (-0·06 and -0·08mm). The 95% limits of agreement were wider for the European/Australian data compared with the East Asian data. Larger deviations between modelled and empirical values were observed at the lower and upper extremes of AL, consistent with increased variability at the tails of the AL distribution (Figure 1, Supplementary Figures 9-12). In the pooled cohort dataset, the

proportion of AL observations that were at or below the 3rd, 15th, 50th, 85th and 97th centiles in the fitted model were 2.94%, 14.56%, 49.63% 84.99% and 97.06%, respectively. The leave-one-cohort-out analysis found the largest changes in centiles with exclusion of the West China Refractive Error Development Survey (MAD=0.079mm), Generation R (MAD=0.039mm) and the Sydney Myopia Study (MAD=0.030mm), which represented the largest, 3rd largest and 4th largest cohorts, respectively. All other MADs were <0.02mm.

© 2026 Kneepkens SCM et al. *JAMA Ophthalmology*.

eDiscussion

To our knowledge this study benefits from the largest and most diverse datasets on childhood ocular biometry to date, combining individual-level data from 16 cohorts across East Asia, Europe, and Australia. The large sample size, geographic coverage, and inclusion of longitudinal measurements enabled detailed modelling of regional and sex-specific growth trajectories. Additional strengths include the robust and repeatable measurement of AL, and the inclusion of recent data reflecting contemporary environments. To assess internal model consistency, we compared the modelled centile values to empirical values derived directly from the pooled dataset. We found excellent agreement, with intraclass correlation coefficients of 0·99 across all sex and regional subgroups. The aim of the centile model is not to fit all empirical data perfectly but to generate biologically plausible, smoothed curves while fitting most empirical data well. Accordingly, the mean bias between modelled and empirical values was minimal across ages: 0·04 mm for Western males and females, and −0·08 mm and −0·06 mm for East Asian males and females, respectively. Deviations between modelled and empirical data are expected as the latter is also subject to sampling error (i.e., is an estimate of the true population). This is demonstrated by the limits of agreement, which were larger for the European/Australian cohort and with larger differences occurring at high and low centiles. These deviations at the extremes reflect greater variability and sparser data for model fitting in the tails of the distribution, where model fits may be less stable but empirical sampling error is also larger (see supplementary Figures 10-12). Accordingly, the centile curves are most reliable in the central range, with increasing uncertainty toward the extremes, also shown in Figure 1. By generating confidence intervals around centiles and using sensitivity analyses, we demonstrated that the current centile model was largely robust to: i) sampling error, ii) model method (vs GAMLSS), and iii) inclusion of both vs one eye data. The leave-one-cohort-out analysis did indicate removing the West China Refractive Error Development Survey had relatively large impact on East Asian centiles (MAD=0.08mm) likely because this study was so large and the sole contributor of data at ages 13 to 18 years in East Asia. In summary our model evaluation

© 2026 Kneepkens SCM et al. *JAMA Ophthalmology*.

demonstrates very good internal consistency for central centiles (15, 50, 85) across ages, sex and regions. There is greater uncertainty in the estimation of more extreme centiles (e.g., 3, 97) and these results should be interpreted accordingly.

© 2026 Kneepkens SCM et al. *JAMA Ophthalmology*.

Section 4: Supplementary figures

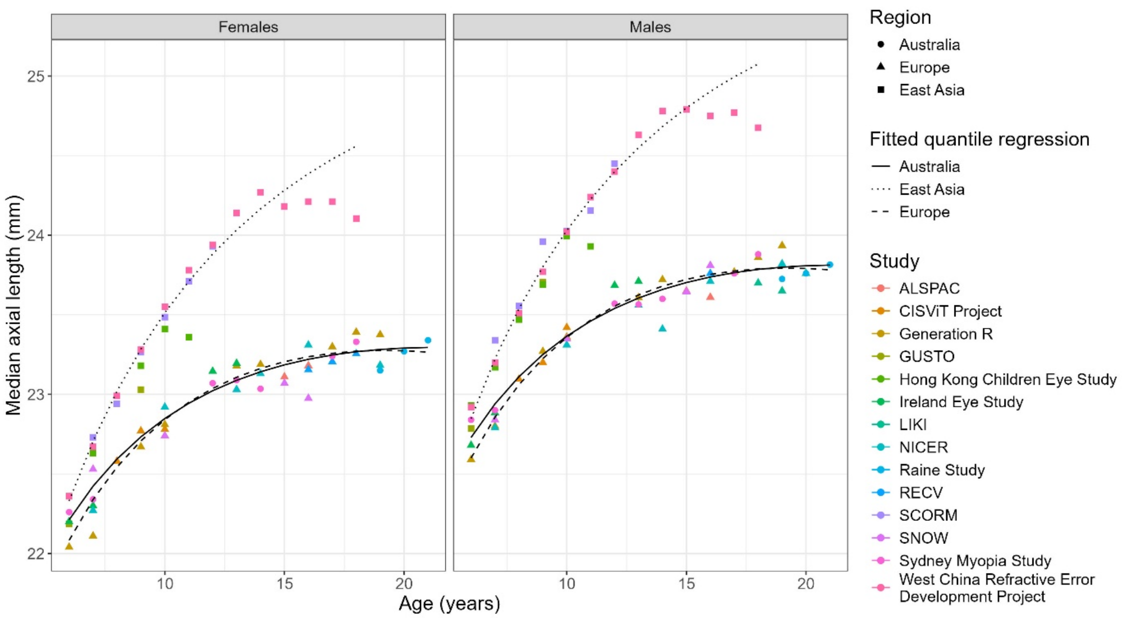

Supplementary Figure 1: Median axial length by region and sex with quantile regression fits (black lines, fit using age and logarithm of age as explanatory variables).

© 2026 Kneepkens SCM et al. *JAMA Ophthalmology*.

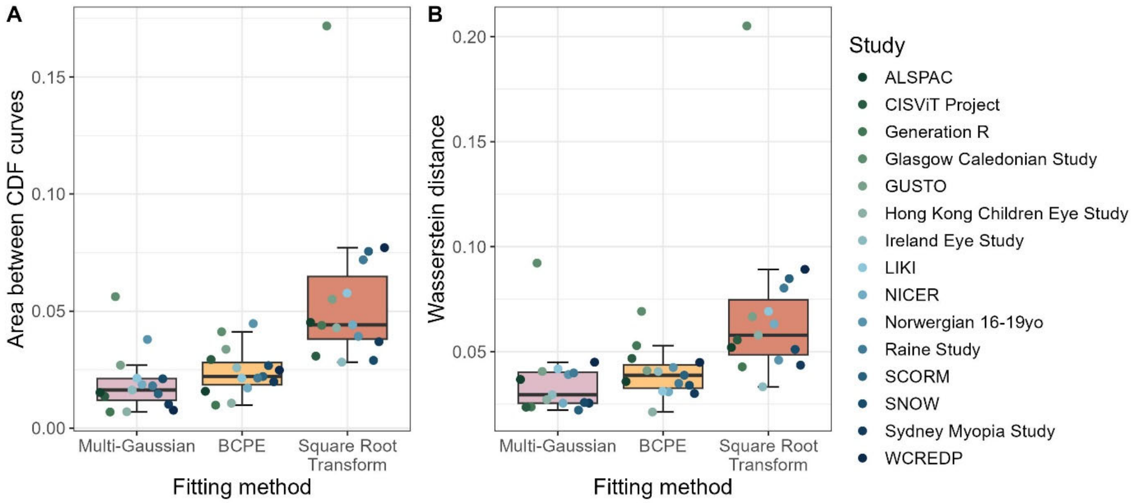

Supplementary Figure 2: Comparison of Multi-gaussian vs Box-Cox Power Exponential (BCPE) vs a square root transformation and normal distribution fit for modelling axial length distribution.

© 2026 Kneepkens SCM et al. *JAMA Ophthalmology*.

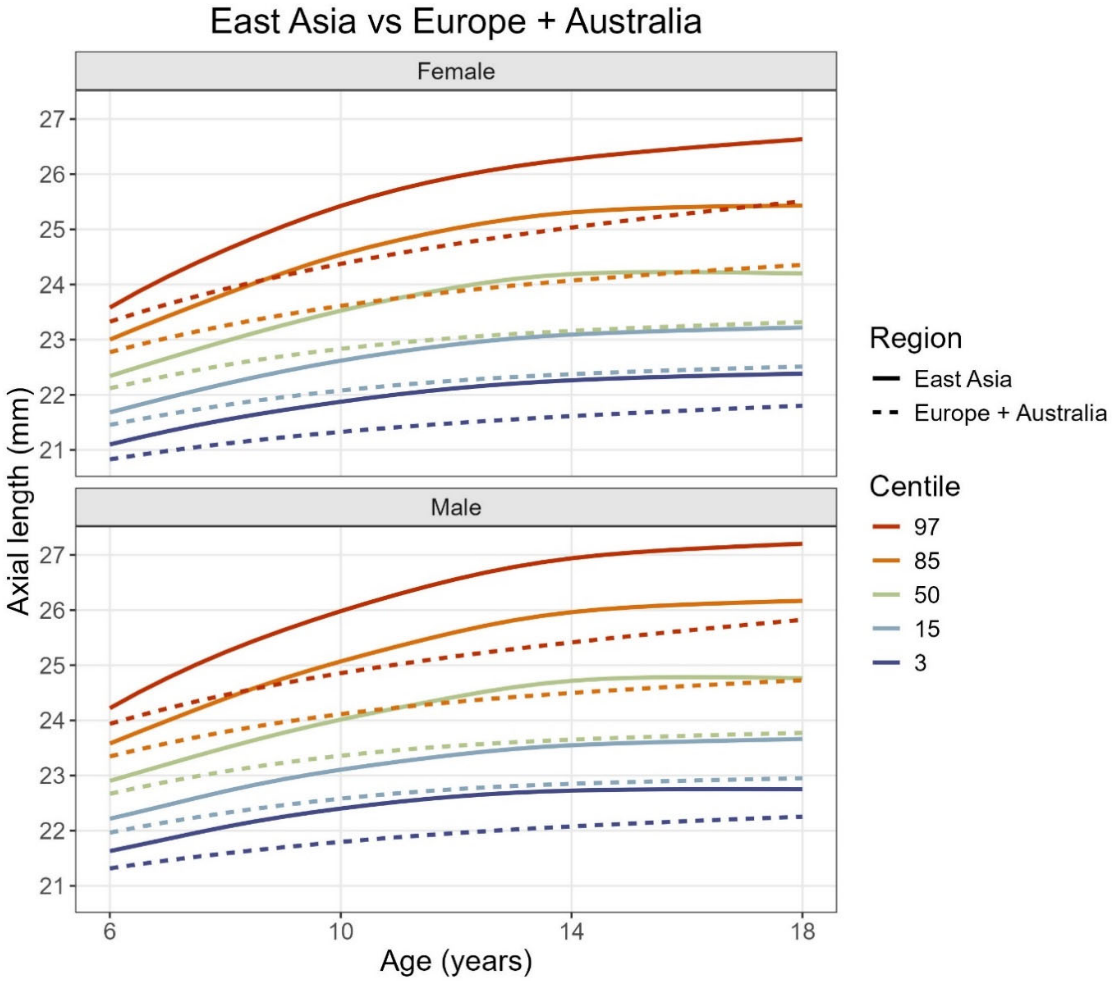

Supplementary Figure 3: Comparison of axial length centile curves from East Asian cohorts (solid lines) and European and Australian cohorts (dashed lines) for males and females.

© 2026 Kneepkens SCM et al. *JAMA Ophthalmology*.

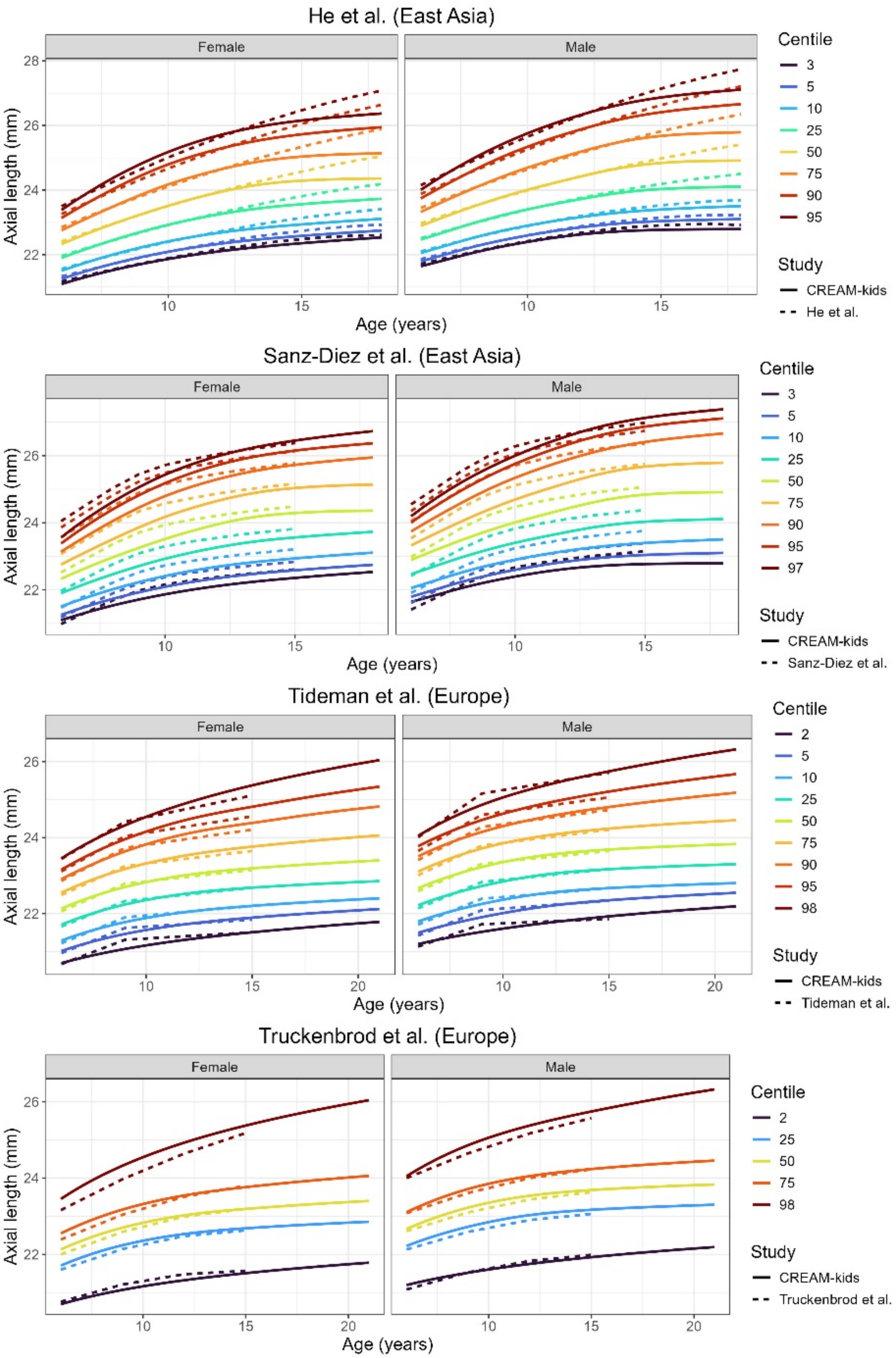

Supplementary Figure 4: Comparison of axial length centiles in the current study (CREAM-Kids) to published studies. Centiles for comparison are based on available published data, with the exception that 1% and 99% are not shown for Sanz-Diez et al. Centile color coding changes across panels.

© 2026 Kneepkens SCM et al. *JAMA Ophthalmology*.

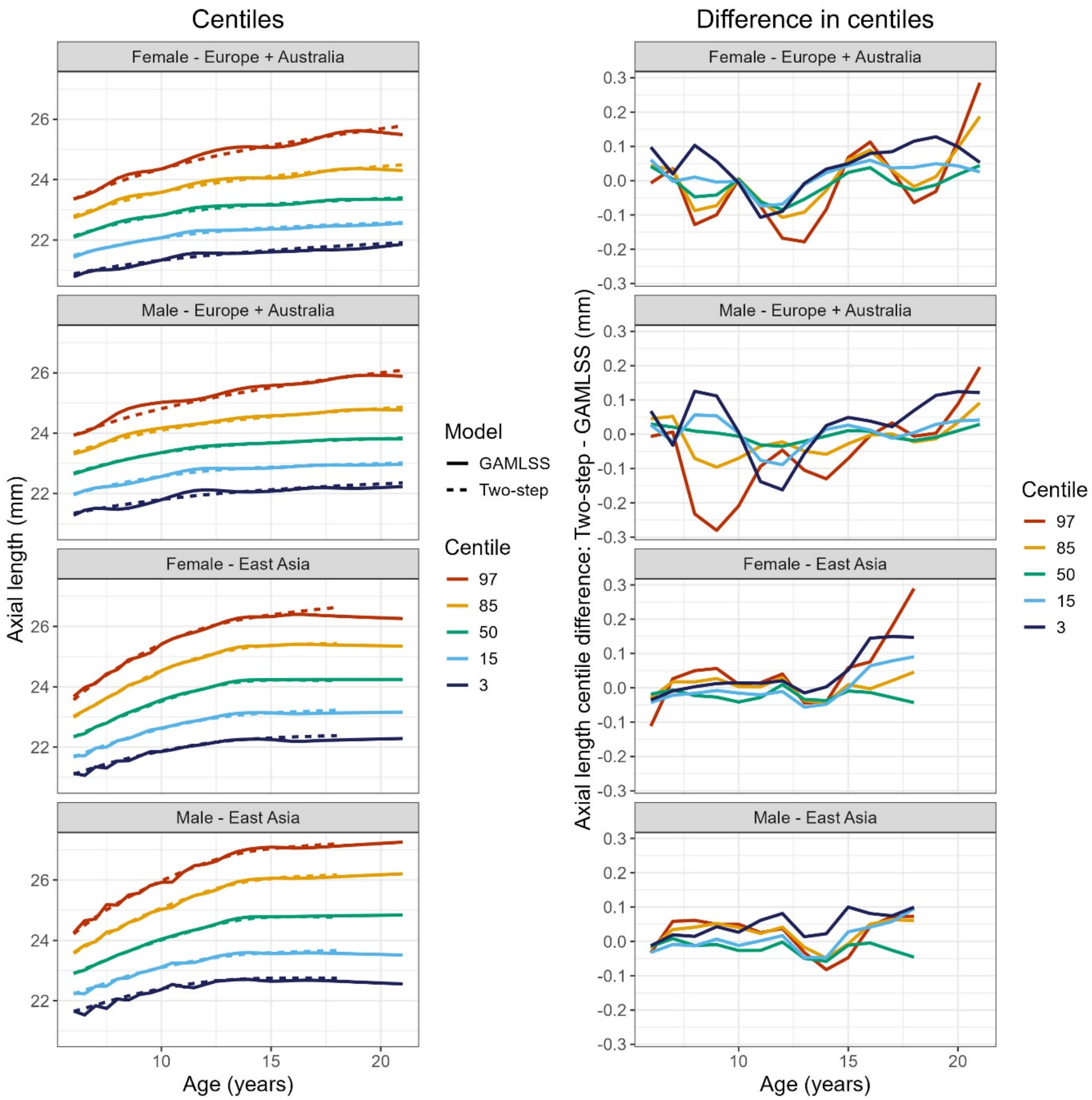

Supplementary Figure 5: Sensitivity analysis comparing the model used in the current study (referred to as a “Two-step” model to a Generalised Additive Model of Location Scale and Shape (GAMLSS) centile model fit using the Box-Cox Power Exponential distribution. Left show the centiles and right show the difference between the two model (i.e., sensitivity model centile value subtracted from main model centile value).

© 2026 Kneepkens SCM et al. *JAMA Ophthalmology*.

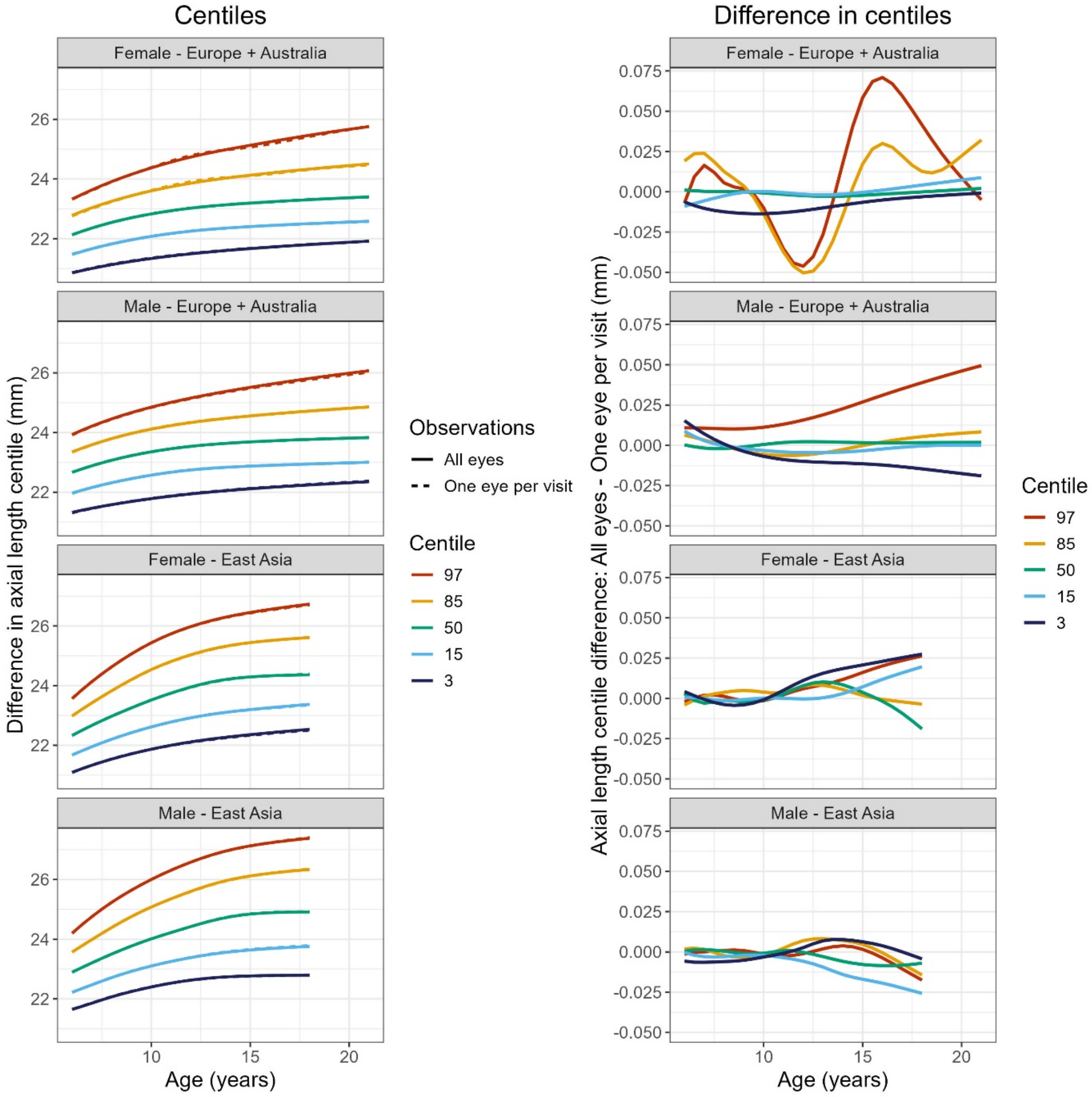

Supplementary Figure 6: Sensitivity analysis comparing the model used in the current study with data from all eyes from all visits with the same model using only data from one eye per visit across all visits. Left show the centiles and right show the difference between the current and GAMLSS model (i.e., sensitivity model centile value subtracted from main model centile value).

© 2026 Kneepkens SCM et al. *JAMA Ophthalmology*.

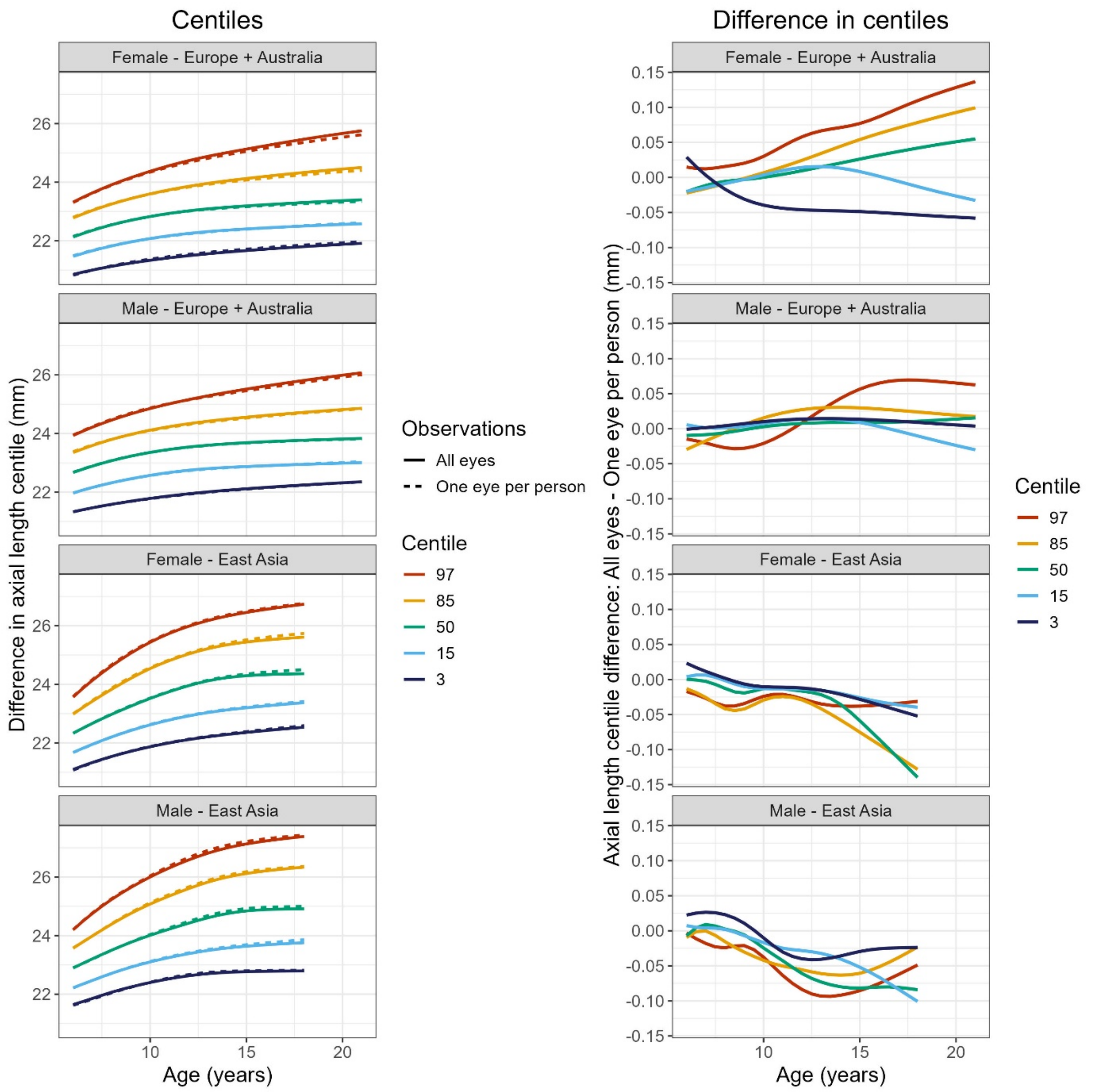

Supplementary Figure 7: Sensitivity analysis comparing the model used in the current study with data from all eyes from all visits with the same model using only data from one eye per person across all visits. Left show the centiles and right show the difference between the current and GAMLSS model (i.e., sensitivity model centile value subtracted from main model centile value

© 2026 Kneepkens SCM et al. *JAMA Ophthalmology*.

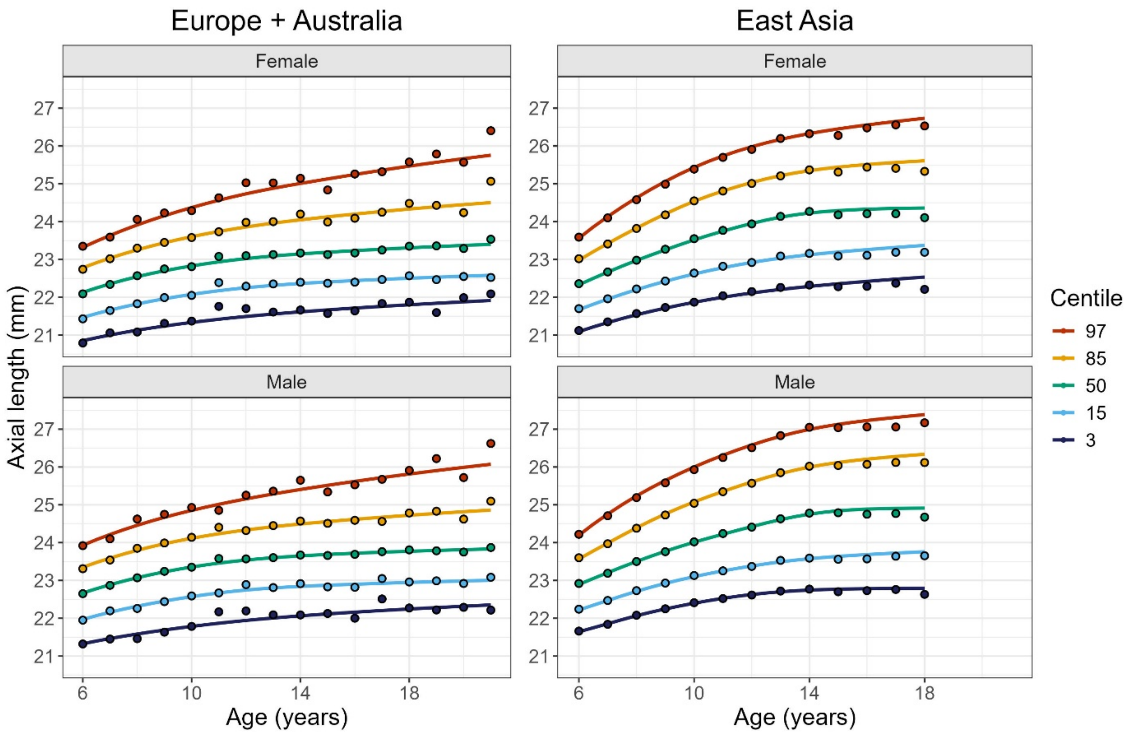

Supplementary Figure 8: A comparison of empirical and model centile values. The points are empirical quantile/centile values drawn from the pooled cohort data (excluding STORM and 43% of Generation R) and clustered by age, sex and region overlayed upon the model centiles at the same age, sex and region (lines). Data are compared only at the 3rd, 15th, 50th, 85th and 97th centiles.

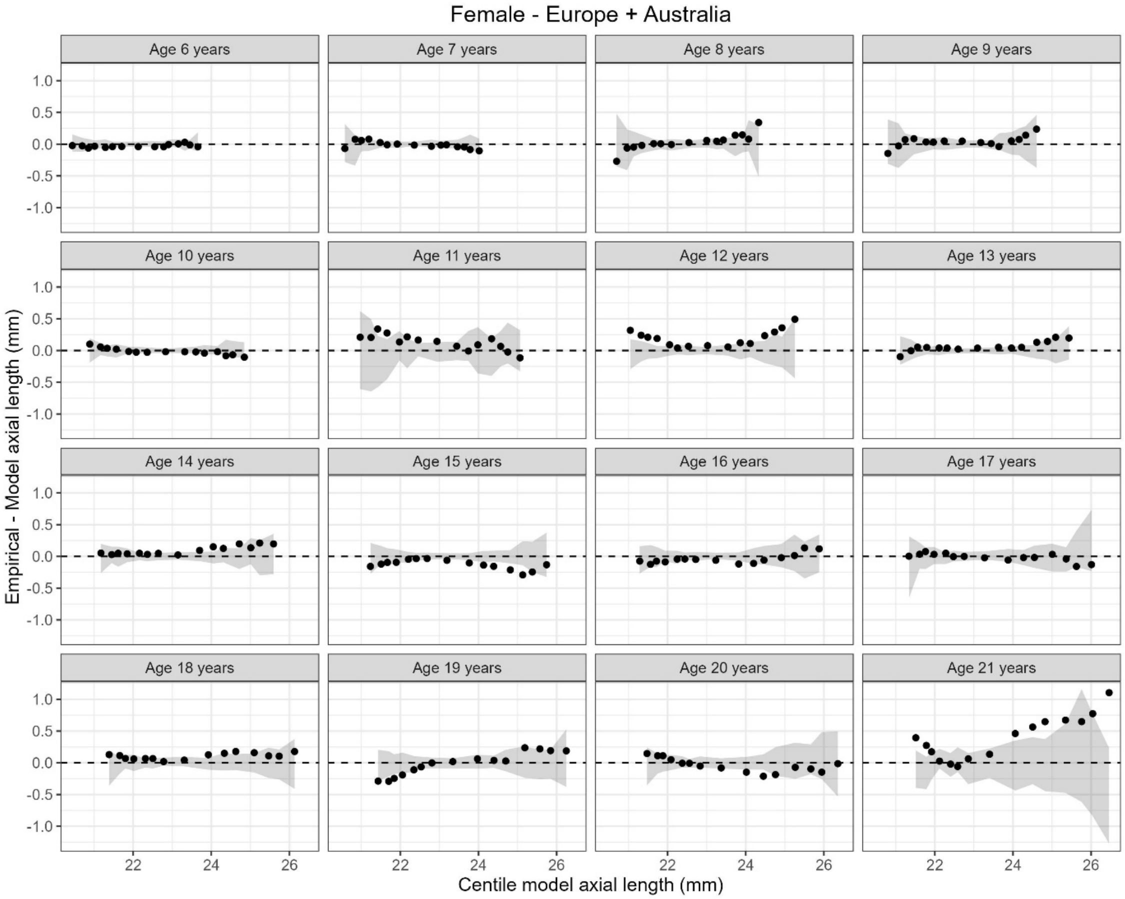

Supplementary Figure 9: Worm plots showing internal model consistency within females in Europe or Australia. Empirical quantiles were extracted from pooled cohort data clustered by age, sex and region at centile values of 1, 2, 3, 5, 10, 15, 25, 50, 75, 85, 90, 95, 97, 98 and 99. Note pooled empirical data does not include 43% of the Generation R cohort. Each point shows the difference in mm between the model and empirical centile value and is plotted against the model centile value to avoid assuming a distribution of the residuals. The shaded are represents the expected sampling error generated by bootstrap resampling of empirical quantile estimates (1000 replicates). The model is more consistent with the empirical data when the points follow the dashed horizontal line indicating a consistent, low bias across the centiles.

© 2026 Kneepkens SCM et al. *JAMA Ophthalmology*.

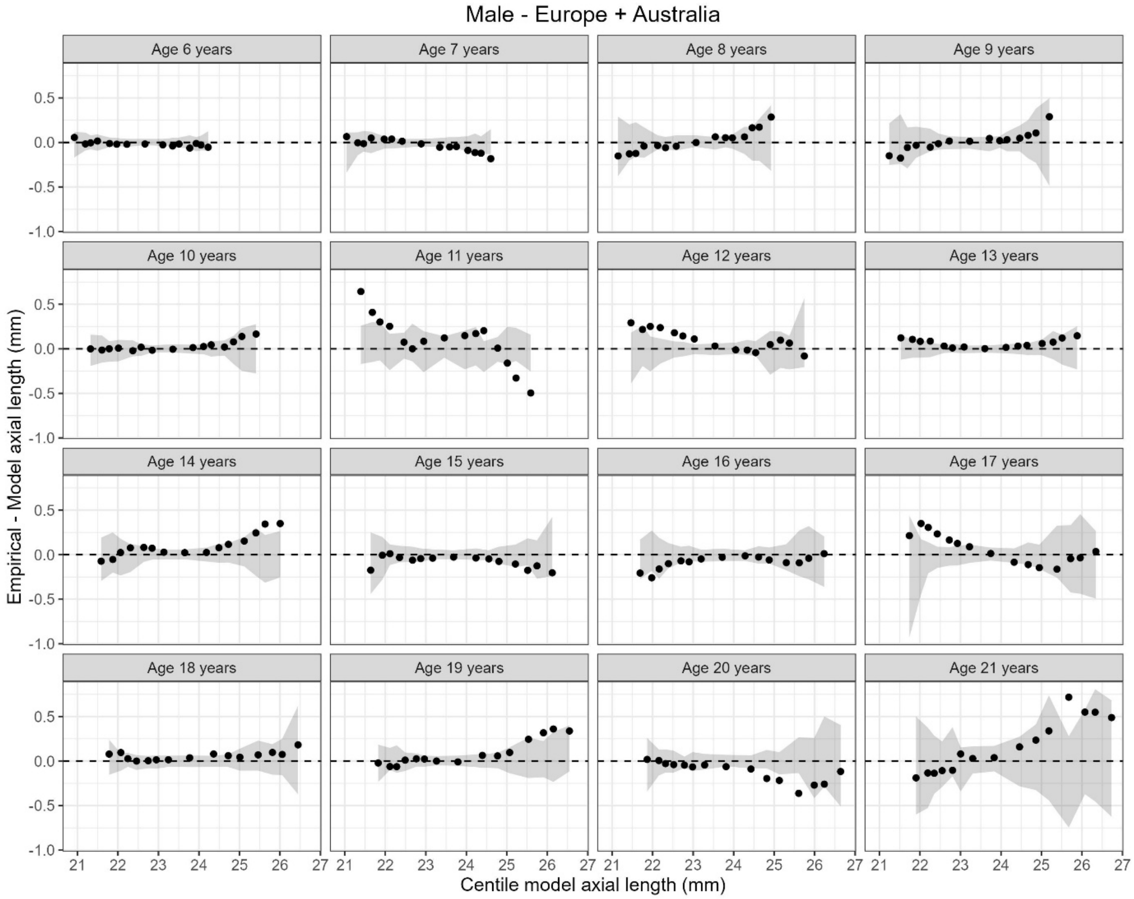

Supplementary Figure 10: Worm plots showing internal model consistency within males in Europe or Australia. Empirical quantiles were extracted from pooled cohort data clustered by age, sex and region at centile values of 1, 2, 3, 5, 10, 15, 25, 50, 75, 85, 90, 95, 97, 98 and 99. Note pooled empirical data does not include 43% of the Generation R cohort. Each point shows the difference in mm between the model and empirical centile value and is plotted against the model centile value to avoid assuming a distribution of the residuals. The shaded are represents the expected sampling error generated by bootstrap resampling of empirical quantile estimates (1000 replicates). The model is more consistent with the empirical data when the points follow the dashed horizontal line indicating a consistent, low bias across the centiles.

© 2026 Kneepkens SCM et al. *JAMA Ophthalmology*.

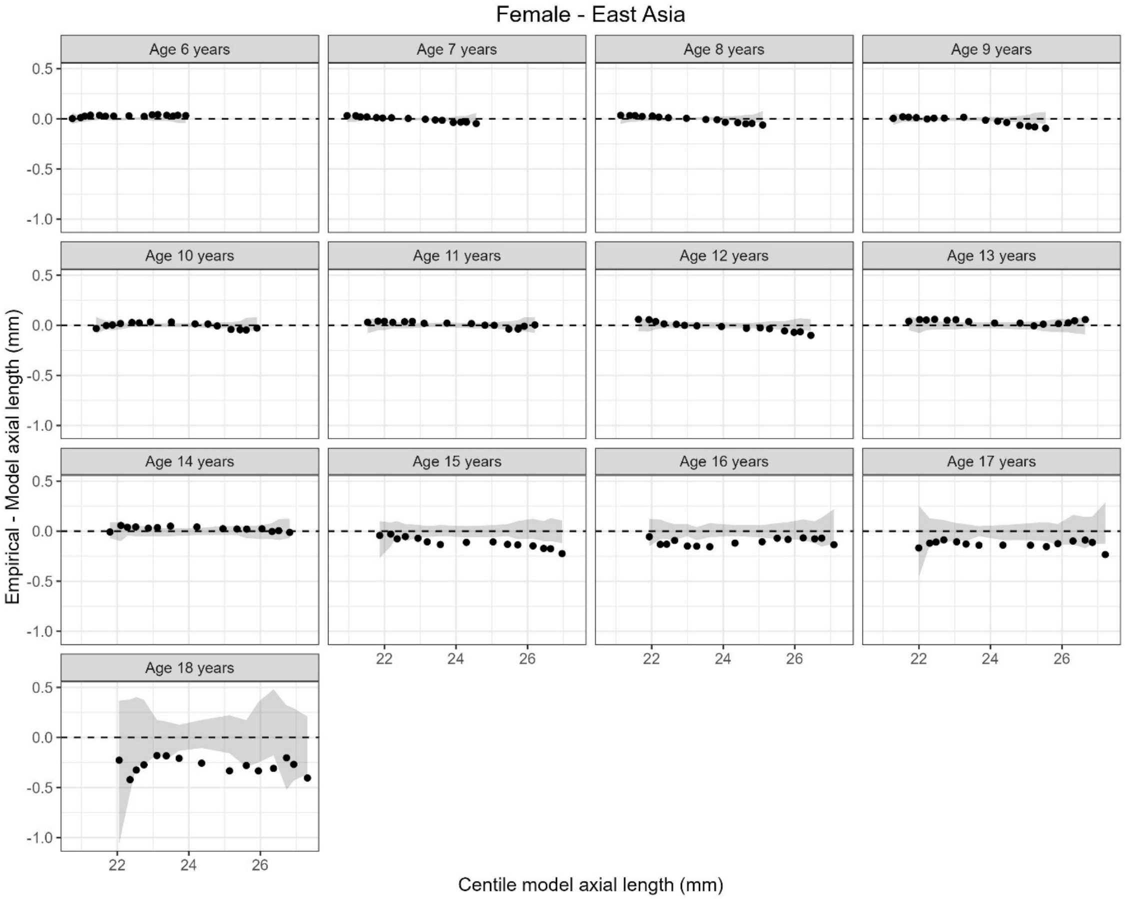

Supplementary Figure 11: Worm plots showing internal model consistency within females in East Asia. Empirical quantiles were extracted from pooled cohort data clustered by age, sex and region at centile values of 1, 2, 3, 5, 10, 15, 25, 50, 75, 85, 90, 95, 97, 98 and 99. Each point shows the difference in mm between the model and empirical centile value and is plotted against the model centile value to avoid assuming a distribution of the residuals. Note pooled empirical data does not include the STORM cohort and likely explains the negative bias at ages 15-18 years. The shaded are represents the expected sampling error generated by bootstrap resampling of empirical quantile estimates (1000 replicates). The model is more consistent with the empirical data when the points follow the dashed horizontal line indicating a consistent, low bias across the centiles.

© 2026 Kneepkens SCM et al. *JAMA Ophthalmology*.

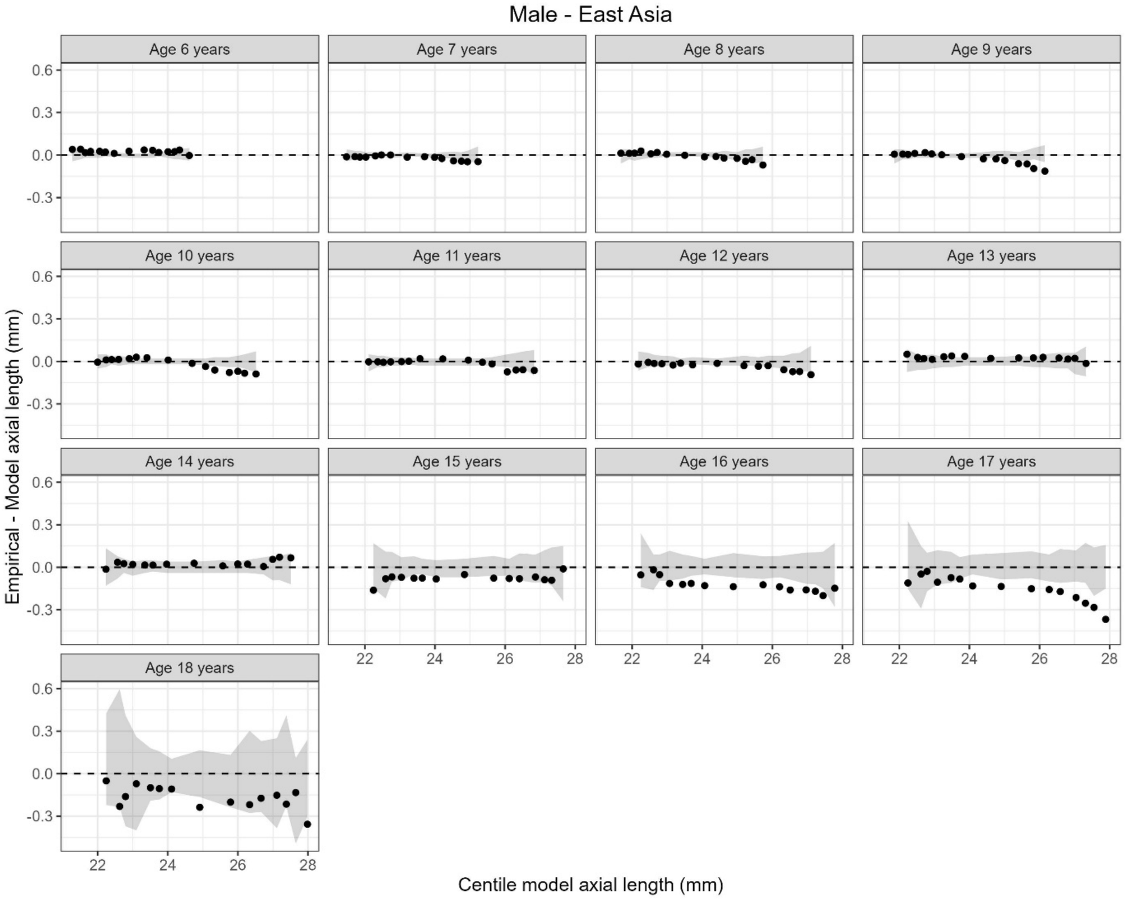

Supplementary Figure 12: Worm plots showing internal model consistency within males in East Asia. Empirical quantiles were extracted from pooled cohort data clustered by age, sex and region at centile values of 1, 2, 3, 5, 10, 15, 25, 50, 75, 85, 90, 95, 97, 98 and 99. Each point shows the difference in mm between the model and empirical centile value and is plotted against the model centile value to avoid assuming a distribution of the residuals. Note pooled empirical data does not include the STORM cohort and likely explains the negative bias at ages 15-18 years. The shaded are represents the expected sampling error generated by bootstrap resampling of empirical quantile estimates (1000 replicates). The model is more consistent with the empirical data when the points follow the dashed horizontal line indicating a consistent, low bias across the centiles.

© 2026 Kneepkens SCM et al. *JAMA Ophthalmology*.

Section 5: Supplemental Tables 1-5

Supplementary Table 1: Leave one out analysis assessing the influence of individual studies within-region variance estimates.

Excluded study	Between-study estimate (%)	Between-Participant estimate (%)	Residual estimate (%)
**Europe + Australia**			
None (pooled)	0·009 (1.21%)	0·66 (87.50%)	0·09 (11.30%)
Generation R	0·005 (0·62%)	0·66 (88.87%)	0·08 (10·51%)
Glasgow Caledonian Study	0·009 (1.23%)	0·66 (88.64%)	0·08 (10·13%)
NICER	0·01 (1.32%)	0·64 (86.17%)	0·09 (12.51%)
Ireland Eye Study	0·009 (1.14%)	0·67 (87.78%)	0·09 (11.08%)
CISViT Project	0·008 (1.10%)	0·66 (87.11%)	0·09 (11.79%)
SNOW	0·01 (1.37%)	0·67 (87.50%)	0·09 (11.14%)
Norwergian 16-19yo	0·009 (1.17%)	0·66 (87.54%)	0·09 (11.29%)
LIKI	0·008 (1.08%)	0·64 (87.41%)	0·09 (11.50%)
**East Asia**			
None (pooled)	0·002 (0·25%)	0·74 (92.54%)	0·06 (7.21%)
West China Refractive Error Development Project	0·007 (0·97%)	0·62 (81.30%)	0·14 (17.73%)
SCORM	0·002 (0·27%)	0·74 (92.69%)	0·06 (7.05%)
GUSTO	0·0006 (0·07%)	0·74 (92.73%)	0·06 (7.20%)
Hong Kong Children Eye Study	0·000006 (0·0008%)	0·75 (92.87%)	0·06 (7.13%)

© 2026 Kneepkens SCM et al. *JAMA Ophthalmology*.

Supplementary Table 2: Number of observations using either persons, visits or eyes as the unit of analysis, used to generate centile models by age, sex and region.

	East Asia				Europe + Australia			
	Female		Male		Female		Male	
Age	Person	Eyes	Persons	Eyes	Persons	Eyes	Persons	Eyes
6	10425	30313	10997	31871	1443	6135	1477	5962
7	11502	40034	12571	43723	1061	2844	1077	2920
8	8849	35899	9755	38970	469	1512	640	1623
9	7500	31756	8198	34493	404	1623	304	1726
10	5103	23942	5595	26054	1098	5569	1100	5369
11	4070	18837	4650	21462	NA	NA	NA	206
12	3402	15213	3772	17142	559	1481	497	1328
13	3001	12710	3358	14484	1332	5040	1521	5100
14	2065	8546	2467	10276	444	3171	588	3145
15	1518	5004	1299	4715	1448	2542	1255	2236
16	1270	4386	1053	3818	773	2069	536	2041
17	801	2771	743	2596	258	1109	346	1015
18	140	466	158	536	417	2533	775	2804
19	NA	NA	NA	NA	236	1679	810	2880
20	NA	NA	NA	NA	572	1144	797	1594
21	NA	NA	NA	NA	NA	NA	97	194
Total	59646	229877	64616	250140	10514	38451	11820	40143

Note number of observations differs from Table 1 as eligible observations were not included in the centile model if there were insufficient (<85) participants in a particular region, sex and age group.

Supplementary Table 3: Axial length centile values for European and Australian males and females across a selected range of centiles

Age	Sex	2	3	5	10	15	25	50	75	85	90	95	97	98
**Europe + Australia - Males**														
6	Male	21.20	21.33	21.49	21.79	21.97	22.22	22.67	23.11	23.35	23.51	23.77	23.93	24.05
7	Male	21.32	21.46	21.65	21.97	22.16	22.42	22.89	23.34	23.59	23.76	24.04	24.21	24.36
8	Male	21.43	21.58	21.79	22.12	22.32	22.58	23.07	23.55	23.80	23.97	24.27	24.46	24.63
9	Male	21.52	21.69	21.91	22.25	22.45	22.72	23.23	23.71	23.97	24.15	24.46	24.67	24.86
10	Male	21.60	21.78	22.01	22.37	22.57	22.85	23.35	23.85	24.11	24.30	24.63	24.85	25.06
11	Male	21.68	21.87	22.11	22.46	22.67	22.95	23.46	23.96	24.23	24.43	24.78	25.01	25.23
12	Male	21.75	21.94	22.19	22.54	22.75	23.03	23.54	24.05	24.33	24.54	24.90	25.16	25.38
13	Male	21.81	22.01	22.25	22.60	22.80	23.09	23.60	24.12	24.42	24.64	25.02	25.29	25.51
14	Male	21.87	22.06	22.31	22.64	22.84	23.13	23.65	24.19	24.49	24.73	25.12	25.41	25.63
15	Male	21.93	22.11	22.35	22.67	22.87	23.17	23.69	24.24	24.56	24.81	25.21	25.52	25.75
16	Male	21.98	22.16	22.39	22.70	22.90	23.20	23.72	24.28	24.62	24.88	25.30	25.62	25.85
17	Male	22.03	22.20	22.43	22.72	22.92	23.22	23.75	24.32	24.67	24.95	25.38	25.72	25.96
18	Male	22.07	22.24	22.46	22.75	22.95	23.25	23.77	24.36	24.72	25.01	25.46	25.81	26.06
19	Male	22.11	22.28	22.49	22.77	22.97	23.26	23.79	24.40	24.77	25.07	25.54	25.90	26.15
20	Male	22.15	22.32	22.52	22.79	22.99	23.28	23.81	24.43	24.82	25.13	25.61	25.99	26.24
21	Male	22.19	22.35	22.55	22.80	23.00	23.30	23.83	24.46	24.86	25.18	25.67	26.07	26.32
**Europe + Australia - Females**														
6	Female	20·70	20·85	21.01	21.29	21.47	21.71	22.13	22.55	22.78	22.91	23.16	23.32	23.45
7	Female	20·84	21.00	21.19	21.48	21.66	21.92	22.35	22.79	23.04	23.18	23.46	23.63	23.78
8	Female	20·96	21.13	21.34	21.64	21.82	22.10	22.54	23.00	23.25	23.43	23.73	23.91	24.07
9	Female	21.07	21.24	21.46	21.78	21.96	22.24	22.70	23.17	23.44	23.64	23.96	24.16	24.32
10	Female	21.16	21.33	21.57	21.89	22.08	22.36	22.83	23.32	23.60	23.82	24.16	24.37	24.55
11	Female	21.25	21.42	21.66	21.98	22.17	22.46	22.94	23.44	23.74	23.98	24.33	24.57	24.75
12	Female	21.32	21.49	21.73	22.05	22.25	22.53	23.02	23.54	23.86	24.11	24.48	24.74	24.93
13	Female	21.39	21.56	21.79	22.11	22.31	22.60	23.09	23.63	23.96	24.22	24.61	24.88	25.09
14	Female	21.45	21.61	21.85	22.16	22.36	22.64	23.15	23.70	24.05	24.31	24.71	25.01	25.24
15	Female	21.51	21.67	21.89	22.20	22.41	22.68	23.19	23.76	24.13	24.39	24.81	25.13	25.38
16	Female	21.56	21.71	21.94	22.24	22.44	22.72	23.23	23.82	24.20	24.47	24.91	25.25	25.50
17	Female	21.61	21.76	21.98	22.28	22.47	22.75	23.27	23.87	24.27	24.55	25.01	25.36	25.62
18	Female	21.66	21.80	22.02	22.31	22.50	22.78	23.31	23.92	24.33	24.63	25.10	25.47	25.74
19	Female	21.70	21.84	22.05	22.34	22.53	22.81	23.34	23.97	24.39	24.70	25.18	25.57	25.84
20	Female	21.74	21.88	22.09	22.37	22.56	22.83	23.37	24.02	24.45	24.76	25.27	25.67	25.94
21	Female	21.78	21.92	22.12	22.40	22.58	22.86	23.40	24.06	24.50	24.82	25.34	25.76	26.04

© 2026 Kneepkens SCM et al. *JAMA Ophthalmology*.

Supplementary Table 4: Axial length centile values for East Asian males and females across a selected range of centiles

Age	Sex	2	3	5	10	15	25	50	75	85	90	95	97	98
**East Asia - Males**														
6	Male	21.51	21.64	21.79	22.05	22.22	22.47	22.89	23.32	23.57	23.74	24.01	24.20	24.33
7	Male	21.71	21.85	22.02	22.30	22.47	22.73	23.21	23.70	23.99	24.19	24.53	24.75	24.93
8	Male	21.92	22.07	22.25	22.53	22.71	22.98	23.50	24.06	24.39	24.62	24.99	25.23	25.42
9	Male	22.09	22.25	22.44	22.73	22.92	23.21	23.77	24.40	24.76	25.01	25.40	25.64	25.84
10	Male	22.24	22.40	22.60	22.90	23.10	23.40	24.01	24.69	25.08	25.34	25.76	26.00	26.19
11	Male	22.36	22.53	22.73	23.04	23.25	23.57	24.22	24.95	25.35	25.63	26.06	26.31	26.51
12	Male	22.46	22.63	22.85	23.17	23.38	23.72	24.42	25.19	25.60	25.88	26.33	26.58	26.78
13	Male	22.52	22.70	22.94	23.27	23.49	23.86	24.61	25.41	25.82	26.10	26.56	26.81	27.01
14	Male	22.56	22.74	23.00	23.34	23.57	23.97	24.75	25.57	26.00	26.28	26.73	26.99	27.19
15	Male	22.59	22.77	23.04	23.40	23.64	24.03	24.84	25.68	26.12	26.41	26.87	27.13	27.33
16	Male	22.61	22.78	23.07	23.44	23.68	24.07	24.89	25.74	26.21	26.51	26.97	27.23	27.45
17	Male	22.62	22.79	23.09	23.47	23.72	24.09	24.91	25.77	26.28	26.59	27.04	27.31	27.55
18	Male	22.63	22.79	23.10	23.50	23.76	24.11	24.91	25.79	26.34	26.66	27.11	27.38	27.65
**East Asia - Females**														
6	Female	20·98	21.09	21.25	21.50	21.67	21.90	22.33	22.75	22.98	23.14	23.38	23.56	23.69
7	Female	21.20	21.33	21.51	21.78	21.95	22.20	22.67	23.14	23.42	23.61	23.93	24.13	24.29
8	Female	21.39	21.54	21.74	22.02	22.20	22.47	22.97	23.52	23.83	24.06	24.41	24.63	24.81
9	Female	21.55	21.71	21.93	22.23	22.42	22.71	23.25	23.86	24.20	24.46	24.82	25.06	25.24
10	Female	21.69	21.87	22.09	22.41	22.62	22.93	23.52	24.18	24.54	24.80	25.18	25.43	25.61
11	Female	21.82	22.00	22.23	22.57	22.78	23.11	23.75	24.43	24.81	25.07	25.48	25.74	25.91
12	Female	21.93	22.11	22.35	22.69	22.92	23.27	23.95	24.65	25.04	25.30	25.72	25.98	26.15
13	Female	22.02	22.21	22.44	22.79	23.04	23.39	24.12	24.83	25.22	25.48	25.90	26.18	26.35
14	Female	22.10	22.29	22.52	22.87	23.12	23.49	24.23	24.96	25.35	25.62	26.05	26.33	26.52
15	Female	22.18	22.36	22.58	22.94	23.20	23.56	24.29	25.04	25.44	25.73	26.15	26.45	26.65
16	Female	22.24	22.42	22.64	23.00	23.26	23.63	24.33	25.09	25.51	25.81	26.24	26.56	26.75
17	Female	22.30	22.48	22.70	23.06	23.32	23.68	24.35	25.12	25.56	25.88	26.31	26.65	26.85
18	Female	22.36	22.53	22.75	23.11	23.37	23.73	24.36	25.14	25.61	25.95	26.37	26.73	26.94

© 2026 Kneepkens SCM et al. *JAMA Ophthalmology*.

Supplementary Table 5: Annualised rates of change in axial length centile values for males and female from East Asia or Europe and Australia

	Males					Females				
Age	3%	15%	50%	75%	97%	3%	15%	50%	75%	97%
	**Europe + Australia**									
7	0·13	0·18	0·21	0·23	0·27	0·14	0·18	0·22	0·24	0·30
8	0·12	0·15	0·18	0·20	0·23	0·12	0·16	0·18	0·21	0·27
9	0·10	0·13	0·15	0·17	0·20	0·11	0·13	0·15	0·18	0·24
10	0·09	0·11	0·12	0·14	0·18	0·09	0·11	0·12	0·15	0·21
11	0·08	0·09	0·10	0·11	0·16	0·08	0·09	0·10	0·13	0·19
12	0·07	0·07	0·08	0·10	0·14	0·07	0·07	0·08	0·11	0·16
13	0·06	0·05	0·06	0·08	0·13	0·06	0·06	0·06	0·10	0·14
14	0·06	0·04	0·04	0·07	0·12	0·06	0·05	0·05	0·09	0·12
15	0·05	0·03	0·04	0·06	0·11	0·05	0·04	0·04	0·08	0·12
16	0·05	0·03	0·03	0·06	0·10	0·05	0·03	0·04	0·07	0·12
17	0·04	0·02	0·03	0·05	0·10	0·04	0·03	0·04	0·07	0·11
18	0·04	0·02	0·02	0·05	0·09	0·04	0·03	0·04	0·06	0·11
19	0·04	0·02	0·02	0·05	0·09	0·04	0·03	0·03	0·06	0·10
20	0·04	0·02	0·02	0·04	0·08	0·04	0·03	0·03	0·06	0·09
21	0·03	0·02	0·02	0·04	0·08	0·04	0·02	0·03	0·05	0·09
Age	**East Asia**									
7	0·21	0·25	0·31	0·41	0·53	0·23	0·27	0·33	0·43	0·55
8	0·21	0·24	0·29	0·40	0·46	0·20	0·24	0·30	0·40	0·48
9	0·17	0·20	0·26	0·35	0·39	0·17	0·21	0·28	0·37	0·42
10	0·15	0·17	0·23	0·31	0·34	0·15	0·19	0·26	0·32	0·35
11	0·12	0·15	0·21	0·27	0·30	0·13	0·16	0·22	0·26	0·29
12	0·09	0·13	0·20	0·25	0·26	0·11	0·13	0·20	0·22	0·23
13	0·06	0·10	0·18	0·21	0·22	0·09	0·11	0·15	0·17	0·18
14	0·04	0·08	0·13	0·16	0·17	0·08	0·08	0·10	0·12	0·14
15	0·02	0·06	0·08	0·11	0·12	0·07	0·07	0·05	0·08	0·12
16	0·01	0·05	0·04	0·08	0·09	0·06	0·06	0·03	0·06	0·10
17	0·01	0·04	0·01	0·07	0·08	0·06	0·06	0·02	0·05	0·09
18	0·00	0·03	0·01	0·06	0·07	0·05	0·05	0·01	0·04	0·08

© 2026 Kneepkens SCM et al. *JAMA Ophthalmology*.
