# Supplementary material for: Global Axial Length Centile Charts
Source: JAMA Ophthalmol. 2026 Jul 16:e262539. Online ahead of print. doi: 10.1001/jamaophthalmol.2026.2539 (PMC13377471; doi:10.1001/jamaophthalmol.2026.2539)
Supplement: Supplement 2. — CREAM-Kids Consortium members [file jamaophthalmol-e262539-s002.pdf]

Supplemental Online Content: Nonauthor Collaborators
*First name, last name, and suffix (if applicable) are required and will appear in PubMed.

| *Group Name(s): Consortium Of Refractive Error And Myopia research in Children | | | | | | | |
|---|---|---|---|---|---|---|---|
| ***First Name and Middle Initial(s)** | ***Last Name** | ***Suffix (eg, Jr, III)** | Academic Degrees | Institution | Location (city, state/province, country) | Role or Contribution, eg, chair, principal investigator | Group (if more than 1 Group listed in the byline) and/or Subgroup (eg, Steering Committee) |
| Fabian | Yii | | | | | | |
| J. Willem L. | Tideman | | | | | | |
| Nuria | Vila-Vidal | | | | | | |
| Jan Roelof | Polling | | | | | | |
| Sara | McCullough | | | | | | |
| Li Lian | Foo | | | | | | |
| Ellen | Svarverud | | | | | | |
| Trine | Langaas | | | | | | |
| Cathy | Williams | | | | | | |
| Cecile | Delcourt | | | | | | |
| Christopher | Hammond | | | | | | |
| Chi Pui | Pang | | | | | | |
| Peilun | Dai | | | | | | |
| Daniel | Ting | | | | | | |
| Kathryn | Rose | | | | | | |
| Katie | Williams | | | | | | |
| Klaus | Nordhausen | | | | | | |
| Marja-Liisa | Franssila | | | | | | |
| Jun | Chen | | | | | | |
| Xun | Xu | | | | | | |
| Li | Yong | | | | | | |
| Marcus | Ang | | | | | | |
| Micheal | Moore | | | | | | |
| Niko | Setälä | | | | | | |
| Wei | Pan | | | | | | |
| Samantha | Lee | | | | | | |
| Virginie | Verhoeven | | | | | | |
| Valldeflors | Viñuela-Navarro | | | | | | |

Supplemental Online Content: Nonauthor Collaborators
*First name, last name, and suffix (if applicable) are required and will appear in PubMed.

| ***First Name and Middle Initial(s)** | ***Last Name** | ***Suffix (eg, Jr, III)** | Academic Degrees | Institution | Location (city, state/province, country) | Role or Contribution, eg, chair, principal investigator | Group (if more than 1 Group listed in the byline) and/or Subgroup (eg, Steering Committee) |
|---|---|---|---|---|---|---|---|
| Wu | Fan | | | | | | |
| Yuzhou | Zhang | | | | | | |
| Jun | Zhou | | | | | | |
| Mariam | El Gharbi | | | | | | |
| Alba | Galdón | | | | | | |
| Joan | Pérez-Corral | | | | | | |
| Jani | Moilanen | | | | | | |
| Wan-Ting | Loke | | | | | | |
| Hanne-Mari S. | Thorud | | | | | | |
| Tina R. | Johansen | | | | | | |
| Cecilie O. | Bjørset | | | | | | |
| Hilde R. | Pedersen | | | | | | |
| Gro | Horgen | | | | | | |
| Stuart J. | Gilson | | | | | | |
