# Supplementary material for: Global Axial Length Centile Charts
Source: JAMA Ophthalmol. 2026 Jul 16:e262539. Online ahead of print. doi: 10.1001/jamaophthalmol.2026.2539 (PMC13377471; doi:10.1001/jamaophthalmol.2026.2539)
Supplement: Supplement 3. — Data sharing statement [file jamaophthalmol-e262539-s003.pdf]

Data Sharing Statement

Kneepkens. Global Axial Length Centile Charts. *JAMA Ophthalmol*. Published July 16, 2026.
doi:10.1001/jamaophthalmol.2026.2539

Data
Data available: No

Additional Information
Explanation for why data not available: Individual-level participant data are not publicly available. Access may be granted through participation in the CREAM-KIDS consortium and completion of required cohort approvals and data use agreements.
